# Recreational football as medicine? A meta-analysis of its effects and feasibility in patients with chronic diseases

**DOI:** 10.3389/fpubh.2026.1869982

**Published:** 2026-10-19

**Authors:** Kun Wei, Gang Chen, Tianle Wang, Bo Wang

**Affiliations:** 1 Department of Physical Education, Henan Sport University, Zhengzhou, Henan, China; 2 School of Physical Education and Sport, Henan University, Kaifeng, Henan, China; 3 Physical Education Department of Nanjing University, Nanjing, China; 4 School of Arts, Beijing Sport University, Beijing, China; 5 School of Sport Science, Beijing Sport University, Beijing, China

**Keywords:** cardiometabolic health, cardiorespiratory fitness, chronic diseases, meta-analysis, recreational football

## Abstract

**Objective:**

To quantitatively synthesise the effects of recreational football interventions on physiological and feasibility-related outcomes in people with chronic diseases.

**Methods:**

PubMed, Web of Science, and the Cochrane Library were searched from inception to 6 February 2026, and additional searches of Scopus and Embase were conducted on 17 August 2026. Controlled trials of recreational football in adolescents and adults with selected chronic disease populations were eligible. Three-level random-effects meta-analyses were performed, with cluster-robust (CR2) variance estimation, clustering on the independent trial, applied to every outcome as a robustness check, and certainty of evidence was assessed using GRADE.

**Results:**

Twenty-two study reports representing 17 unique trials (15 randomised and 2 non-randomised controlled trials; 588 unique participants) were included. Recreational football was associated with significant improvements in body fat percentage (*k* = 13; SMD = −0.36, 95% CI − 0.57 to −0.14; *p* < 0.01), muscle mass (*k* = 12; SMD = 0.22, 95% CI 0.01 to 0.44; *p* = 0.05), diastolic blood pressure (*k* = 12; SMD = −0.53, 95% CI − 0.96 to −0.09; *p* = 0.02), mean arterial pressure (*k* = 7; SMD = −0.89, 95% CI − 1.48 to −0.29; *p* = 0.01), resting heart rate (*k* = 10; SMD = −1.01, 95% CI − 1.72 to −0.31; *p* = 0.01), VO₂peak (*k* = 16; SMD = 0.70, 95% CI 0.37 to 1.03; *p* < 0.01), Yo-Yo Intermittent Endurance Test level 1 performance (*k* = 4; SMD = 1.43, 95% CI 0.83 to 2.03; *p* < 0.01), triglycerides (*k* = 11; SMD = −0.56, 95% CI − 0.91 to −0.22; *p* < 0.01), total cholesterol (*k* = 11; SMD = −0.69, 95% CI − 1.08 to −0.30; *p* < 0.01), plasma osteocalcin (*k* = 4; SMD = 1.07, 95% CI 0.53 to 1.60; *p* < 0.01), and P1NP (*k* = 4; SMD = 1.28, 95% CI 0.22 to 2.34; *p* = 0.03). Borderline effects that were not retained under cluster-robust inference were observed for fat mass (SMD = −0.30; *p* = 0.05), systolic blood pressure (SMD = −0.60; *p* = 0.05) and LDL (SMD = −0.50; *p* = 0.05). No statistically significant pooled effects were observed for body weight (SMD = −0.29; *p* = 0.05), BMI (SMD = −0.06; *p* = 0.71), waist circumference (SMD = −0.48; *p* = 0.18), HbA1c (SMD = −0.28; *p* = 0.10), fasting glucose (SMD = −0.11; *p* = 0.48), fasting insulin (SMD = 0.07; *p* = 0.73), HDL (SMD = 0.10; *p* = 0.70), CRP (SMD = −0.88; *p* = 0.13), DXA-derived leg and whole-body bone mineral density or content (all *p* ≥ 0.11), or CTX-1 (SMD = 0.54; *p* = 0.19). Effects on body fat percentage, fat mass, muscle mass, SBP and LDL were sensitive to the assumed pre–post correlation. Under cluster-robust (CR2) inference the statistical interpretation was unchanged for 22 of the 27 outcomes; fat mass, SBP, LDL and P1NP were no longer significant (all p between 0.05 and 0.07) and whole-body bone mineral density became significant (*p* = 0.03). Certainty of evidence was low to very low for most outcomes, and adverse events were summarized narratively because quantitative synthesis was not possible. Mean attendance was 82.7% across 13 independent trials, and mean attrition was 15.6% across 14 independent trials.

**Conclusion:**

Across the selected chronic disease populations represented in the current evidence base, recreational football showed signals of potential benefit for cardiorespiratory fitness and selected cardiovascular, lipid, and bone-related outcomes, although adiposity- and bone-formation-related effects were less robust in sensitivity analyses. However, these findings should be interpreted cautiously due to heterogeneity and generally low or very low certainty of evidence. Recreational football may be considered a supervised adjunctive exercise option.

**Systematic review registration:**

https://www.crd.york.ac.uk/PROSPERO/view/CRD420261365103, Identifier CRD420261365103.

## 1 Introduction

Chronic non-communicable diseases accounted for approximately 43 million deaths in 2021, representing about 75% of non-pandemic global mortality, with a disproportionate share occurring prematurely and in low- and middle-income countries (1, 2). Physical inactivity is one of the most modifiable contributors to this burden. It is estimated to cause 6 to 10% of the major non-communicable diseases worldwide and to shorten population life expectancy (3). Consistent with current disease-specific guidelines, regular physical activity and structured exercise are recommended as core components of chronic disease management, including hypertension, diabetes, and cancer survivorship, with exercise prescription individualized according to clinical status, treatment burden, and functional capacity (4, 5).

Despite this consensus, long-term adherence to structured endurance or resistance training remains modest in clinical populations, and the translational bottleneck for exercise-as-medicine lies less in efficacy than in acceptability, enjoyment, and sustainability (6, 7). Exercise modalities that can deliver a physiologically adequate stimulus while supporting sustained engagement across patients with heterogeneous diagnoses, treatment burdens, and functional limitations are therefore of particular clinical interest (8, 9).

Recreational football, typically small-sided, non-competitive, group-based play, has emerged as one such modality. Activity-profile and physiological analyses show that small-sided games elicit heart rates of 80 to 90% of maximum, repeated accelerations, decelerations, changes of direction, jumps, and kicking actions, producing a concurrent cardiovascular, neuromuscular, and osteogenic load within a single session (10, 11). This multimodal signature distinguishes recreational football from continuous aerobic training and from isolated resistance exercise (12). The format is also socially structured, and the observational and qualitative literature suggests that peer context, team affiliation, and enjoyment can support adherence in sedentary and clinical adults, although acceptability depends on diagnosis, supervision, and training dose (13, 14).

The accumulated experimental evidence is now substantial. Randomized trials in untrained men and women have reported improvements in VO₂max, blood pressure, body composition, and indices of vascular and bone health after 12 to 64 weeks of small-sided football training (15, 16). Meta-analyses have quantified favorable effects on cardiorespiratory fitness, broad-spectrum physical fitness, and bone mineral density, content, and turnover, particularly at mechanically loaded lower-limb sites (17, 18). These findings underpin the “Football is Medicine” platform, which frames recreational football as a potentially scalable health-enhancing intervention across preventive and therapeutic settings (7).

Evidence in chronic disease populations has grown in parallel, with controlled trials now available in hypertension, type 1 and type 2 diabetes, prostate and breast cancer (19–21). The most recent systematic synthesis in this space concluded that recreational football may be beneficial across several non-communicable disease categories but combined results narratively, without pooled estimates across the multidomain outcomes that guide clinical decision-making (22). Although the available studies included clinically heterogeneous populations, they evaluated a broadly comparable recreational football intervention and reported common physiological outcomes across body composition, cardiovascular and cardiorespiratory, metabolic, and bone-health domains. This provided a basis for estimating average effects across the selected chronic disease populations represented in the available evidence, while recognizing that responses may differ according to diagnosis and clinical context. Whether the fitness, cardiometabolic, and musculoskeletal effects reported across these diverse clinical populations translate consistently across chronic disease cohorts, once heterogeneity in diagnosis, age, medication, baseline fitness, and programme design is accounted for, remains unclear. The certainty of evidence supporting each outcome, and the feasibility-related outcomes that determine real-world applicability, have not previously been assessed together within a chronic disease-restricted evidence base.

We therefore conducted a systematic review and three-level meta-analysis of controlled trials of recreational football in selected chronic disease populations, with the primary objective of estimating average pooled effects across the populations represented in the available evidence rather than establishing diagnosis-specific therapeutic effects. Specifically, we aimed to (i) pool effects across body composition, cardiovascular and cardiorespiratory, lipid, glycaemic, and bone outcomes; (ii) characterize between-setting variability using prediction intervals and test the robustness of the pooled estimates to cluster-robust variance estimation; (iii) rate the certainty of evidence with GRADE; and (iv) summarize feasibility-related outcomes.

## 2 Methods

This meta-analysis was conducted and reported following the 2020 PRISMA (Preferred Reporting Items for Systematic Reviews and Meta-Analyses) guidelines (23). Additionally, the study was pre-registered in the PROSPERO database (registration ID: CRD420261365103) to enhance research transparency and objectivity.

### 2.1 Search strategy

Electronic searches were conducted in PubMed, Web of Science, and the Cochrane Library from inception to 6 February 2026 without date restrictions. An additional search of the Scopus and Embase database were conducted on 17 August 2026 to further improve the comprehensiveness of the literature search. A broad search strategy centered on recreational football/soccer terminology was used to maximize sensitivity, because relevant clinical studies are not consistently indexed by population or outcome terms alone. The full search strategy is provided in Supplementary Table S1. No database-level language limits were imposed, but studies were eligible only if titles and abstracts were available in English. Reference lists of included studies and relevant reviews were hand-searched, forward citation tracking was undertaken, and related-record functions were used to identify additional reports. PROSPERO and the Cochrane Database of Systematic Reviews were also checked for ongoing or related reviews.

### 2.2 Eligibility criteria

Eligibility criteria were structured according to the PICOS framework (23). Eligible populations were participants with chronic diseases, including cancer, cardiovascular disease or hypertension, diabetes, and related chronic or metabolic disorders, irrespective of sex, age, baseline fitness, or disease severity, provided that outcome data were reported separately for the clinical population of interest (9, 22). The review was designed to evaluate recreational football as a common exercise-based intervention across selected chronic disease populations rather than to establish diagnosis-specific treatment effects; therefore, studies were considered together when they assessed comparable physiological outcomes. Eligible interventions were recreational football programmes, defined as non-competitive, small-sided, group-based football/soccer activities prescribed primarily for health promotion or fitness improvement rather than formal competition (13, 24). Eligible comparators were non-exercise control, waiting-list control, placebo, usual care, or lifestyle advice without a structured exercise programme. Eligible outcomes included at least one quantitative postintervention measure of body composition, cardiovascular or cardiorespiratory health, metabolic health, or bone health, with sufficient information for effect-size estimation. Eligible study designs were longitudinal controlled intervention studies, including randomized and non-randomized controlled trials, that reported preintervention and postintervention data for both intervention and comparator groups (25). Studies were excluded if they lacked a control group, did not isolate data for eligible chronic disease populations, investigated non-football interventions, or were reviews, conference abstracts, grey literature, case reports, qualitative studies, or other non-original reports. Where multiple publications originated from the same trial or participant cohort, they were identified and treated as linked reports. Complementary outcome data from different reports were retained where relevant, whereas duplicate outcome data and overlapping participants were not counted more than once within the same analysis.

### 2.3 Selection process

Retrieved records were deduplicated in EndNote X9. Two reviewers independently screened titles and abstracts against the eligibility criteria and then assessed full texts for final inclusion. Disagreements were resolved through discussion and, when necessary, adjudication by a third reviewer.

### 2.4 Data extraction

Data extraction was conducted independently by two reviewers using a customized Microsoft Excel worksheet prepared before full-text review, with cross-checking by a third reviewer and consensus resolution involving a fourth reviewer when required. Extracted data included study identifiers, design characteristics, participant characteristics, intervention and comparator details, and outcome data. When numerical data were missing or only presented graphically, study authors were contacted and graphical extraction was performed with WebPlotDigitizer version 4.1 where necessary. Studies were retained for quantitative synthesis only when sufficient numerical information could be obtained. For each group, change scores were calculated as postintervention minus preintervention means, and pooled effects were based on between-group differences in change scores (26, 27). When change-score standard deviations were not reported, they were estimated from baseline and postintervention standard deviations using the following formula:

$$SD_{\text{change}}=\sqrt{S{D^{2}}_{\mathrm{pre}}+S{D^{2}}_{\text{post}}-(2\times r\times SD_{\mathrm{pre}}\times SD_{\text{post}})}$$

A pre–post correlation coefficient of *r* = 0.85 was assumed when deriving change-score standard deviations in the primary analysis (26); the sensitivity of the results to this assumption was examined as described under Statistical analysis.

### 2.5 Risk of bias and methodological quality

Risk of bias in randomized trials was assessed using RoB 2, which evaluates bias arising from the randomization process, deviations from intended interventions, missing outcome data, measurement of the outcome, and selection of the reported result (28). Non-randomized controlled studies were appraised using ROBINS-I (25).

### 2.6 Statistical analysis

All meta-analytic procedures were conducted in R version 4.2.1 using the metafor (27), clubSandwich (29), metaviz and orchaRd packages. Standardized mean differences (SMDs) were computed from the between-group difference in change scores with the escalc() function of metafor (measure = “SMD”), which applies the small-sample correction and therefore returns Hedges’ *g*; effects were interpreted as trivial (< 0.20), small (0.20–0.49), moderate (0.50–0.79), or large (≥ 0.80).

Because some studies contributed more than one effect-size estimate to the same outcome (multiple intervention arms compared against a shared control group, linked reports from the same participant cohort, or more than one eligible measure of the same outcome), effect sizes were treated as nested within trials rather than averaged. Every report was first assigned to the underlying trial or participant cohort from which it originated, so that linked reports of the same trial share a single identifier while genuinely independent trials remain separate irrespective of authorship; the 22 included reports correspond to 17 independent trials, and the report-to-trial assignment is given in Supplementary Table S11. For each outcome, a three-level random-effects model was fitted with the rma.mv() function, specifying random = ~ 1 | trial/effect, where trial identifies the independent trial or cohort and effect identifies each effect-size estimate within it. The model therefore contains three variance components: the known sampling variance of each estimate (level 1), the variance among effect sizes within trials, σ^2^₂ (level 2), and the variance among trials, σ^2^₃ (level 3). For multi-arm studies, separate effect sizes were calculated for each eligible intervention–control comparison without splitting the shared comparator sample size. Because the sampling covariance among effect sizes from the same trial is not reported in primary studies, a working variance–covariance matrix V was constructed with clubSandwich:impute_covariance_matrix(), blocked by trial and assuming a correlation of ρ = 0.60 among the sampling errors of effect sizes from the same trial; this ρ is distinct from the pre–post correlation r used to derive change-score standard deviations. This value lies mid-range among those conventionally assumed when the correlation cannot be recovered from the primary reports (29, 30) and lies within the range of 0.47 to 0.67 (median 0.50) implied by the Gleser and Olkin (31) expression for effect sizes that share a control group in the five trials that contribute more than one effect size to a single outcome (Supplementary Table S9). Ten of the 27 outcomes contain no within-trial dependence at all, in 16 outcomes a single trial contributes two effect sizes, and only for VO₂peak do four trials do so (Supplementary Table S10), so the influence of ρ remains limited; it was nevertheless varied across ρ = 0.20, 0.40, 0.60 and 0.80 under both inferential procedures (Supplementary Table S8). This matrix was supplied as the V argument of rma.mv (), so that each effect size was weighted by the inverse of its total variance (sampling variance plus the estimated within- and between-study components) while allowing for correlated sampling errors within studies. Variance components were estimated by restricted maximum likelihood (method = “REML”). Confidence intervals and *p*-values for the pooled effect were based on the t distribution with k − 1 degrees of freedom (test = “t”), which is more conservative than the default normal approximation when the number of estimates is small (27). Because the construction of a working covariance matrix is not itself a robust variance estimator, every outcome model was additionally refitted with cluster-robust (sandwich) variance estimation, using the bias-reduced CR2 estimator with the trial as the clustering variable and Satterthwaite small-sample degrees of freedom, implemented with robust (…, clubSandwich = TRUE) (29, 32). Model-based and cluster-robust results are reported side by side for every outcome in Supplementary Tables S6, S7, and conclusions were drawn only where the two procedures agreed. Model-based inference was retained as the primary analysis because the Satterthwaite degrees of freedom fell below the value of four recommended for reliable CR2 inference in 8 of the 27 models (32). Throughout, k denotes the number of effect-size estimates and “Trials” the number of contributing independent trials in each model; for outcomes in which each trial contributed a single estimate, σ^2^₂ is not estimable and the model reduces to a conventional random-effects model.

Heterogeneity was characterized with Cochran’s Q and with the multilevel *I*^2^, which apportions the total heterogeneity into its within-trial (level 2) and between-trial (level 3) components (33, 34), together with τ^2^ and τ; 95% prediction intervals, obtained with predict(), described the expected distribution of true effects across comparable settings (35–37). Statistical power for each pooled estimate was estimated and displayed with sunset (power-enhanced funnel) plots using the metaviz package (38). Potential moderators were explored through subgroup analyses and meta-regression (rma.mv() with the moderator specified in mods, retaining the same random-effects structure), restricted to categories represented by at least five studies (39). Pooled estimates were visualized as orchard plots with the orchaRd package.

Four sets of sensitivity analyses were conducted. First, to examine the influence of the assumed pre–post correlation, change-score standard deviations were re-derived under *r* = 0.20, 0.50, and 0.80 and every outcome-specific model was refitted; a finding was regarded as robust when its direction and statistical interpretation were unchanged across all four assumptions under both inferential procedures. Second, leave-one-out analyses were performed at the trial level: effect sizes from the same trial were first combined into one trial-level estimate with the aggregate() function of metafor, using the covariance implied by the fitted three-level model, and a random-effects model (REML) was then refitted on these trial-level estimates after omitting each trial in turn (leave1out()); because these analyses operate on trial-level estimates, their heterogeneity statistics are not directly comparable with the multilevel *I*^2^ of the primary models. Third, non-randomized controlled trials were retained in the primary analysis because they met all eligibility criteria and contributed comparative data, thereby preserving information from a limited evidence base; because the absence of random allocation may nevertheless introduce confounding and selection bias, outcome models were refitted after excluding these trials, restricted *a priori* to the outcomes to which at least two non-randomized trials contributed (Supplementary Table S10). Fourth, small-study effects were explored with contour-enhanced funnel plots and Egger’s regression test (regtest()) applied to the trial-level estimates obtained as above, recognizing that these procedures are underpowered and unstable when based on small numbers of studies (40).

Mean differences (MDs) were additionally pooled for outcomes measured on a common, clinically interpretable scale, using the same model specification with measure = “MD.”

### 2.7 Certainty of the evidence

Certainty of evidence for each pooled outcome was rated with the GRADE approach as high, moderate, low, or very low (41). Judgments considered risk of bias, inconsistency, indirectness, imprecision, and publication bias, and were made independently by two reviewers with consensus resolution of disagreements (42).

## 3 Results

### 3.1 Study selection

The database search conducted on 6 February 2026 yielded 1,364 records. After removal of 261 duplicates, 1,103 records underwent title and abstract screening, and two additional records were identified through backward citation tracking. Following full-text assessment of 205 reports, 22 studies met the inclusion criteria (20 identified through database searching and two through other methods). Additional searches of Scopus and Embase were conducted on 17 August 2026, identifying 163 and 220 records, respectively. All 383 records were excluded after title and abstract screening, and no additional eligible studies were identified. Therefore, the final number of included studies remained 22 (19, 21, 43–62). The study selection process is shown in Figure 1.

**Figure 1:**
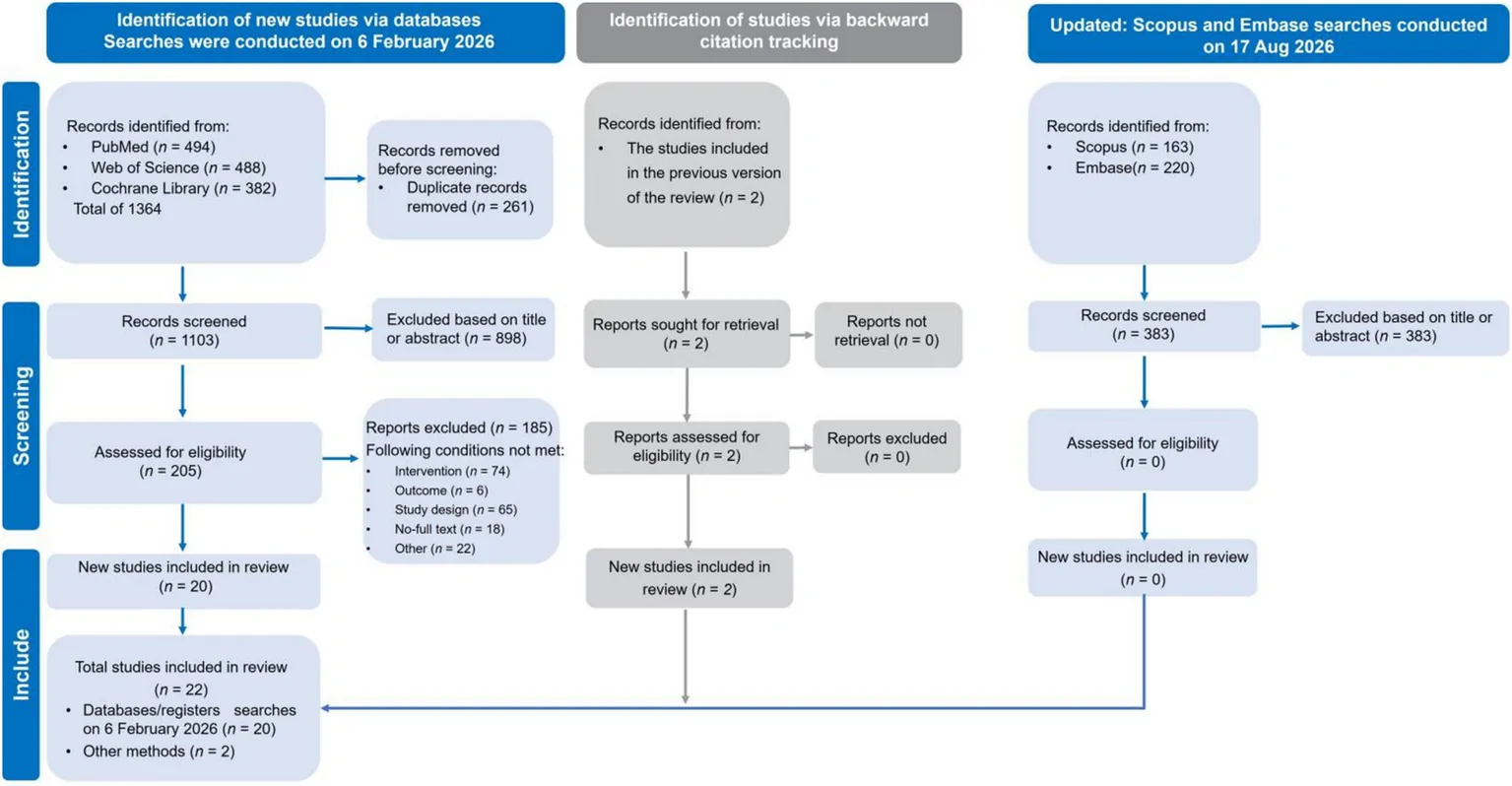
PRISMA flow diagram for included and excluded studies.

### 3.2 Characteristics of included studies

The 22 study reports represented 17 unique trials or participant cohorts (15 randomized and 2 non-randomized controlled trials), comprising 588 unique participants, of whom 315 were allocated to football-based intervention groups and 273 to comparator groups. Based on the reported sex distribution, approximately 374 participants were male, 165 were female, and sex was unclear for 49 participants. Included populations were predominantly clinical cohorts with hypertension, prediabetes, type 1 diabetes, type 2 diabetes, breast cancer, prostate cancer, or broader chronic or metabolic risk profiles. Mean age ranged from 13.9 to 67.1 years, and baseline BMI, when reported, ranged from 19.9 to 33.4 kg·m^−2^. Intervention duration ranged from 8 weeks to 12 months, and programmes were typically delivered two to three times per week, with only one study using a once-weekly format. Exercise intensity was reported in 14 study entries and was generally in the moderate-to-vigorous range; 18 entries reported net playing time (7 to 45 min) and 21 reported total session duration (24 to 90 min). Baseline study and participant characteristics are summarized in Table 1, and intervention characteristics are presented in Table 2.

**Table 1:** Characteristics of the included studies.

Author	Group	Type	N	Males (%)	Age	Height	Weight	BMI	Comorbidity
Teixeira et al. (61)	SSG	RCT	20	100	48.0	n/a	n/a	32.2	Hypertension
	CON		19	100	51.0	n/a	n/a	32.0	
Poffé et al. (59)	SSG	RCT	15	93.3	63.7	174.0	81.8	27.0	Hypertension(most cases)
	CON		18	94.4	63.2	175.9	81.4	26.3	
Mohammed et al. (60)^a^	SSG	RCT	10	100	17.8	156.0	58.2	23.7	Type 1 diabetes
	SDG		10	100	14.5	166.0	55.6	19.9	
	DG		10	100	15.6	157.0	53.2	21.7	
	CON		10	100	14.4	161.0	61.1	23.5	
Soares et al. (58)	SSG	RCT	13	61	60.5	n/a	87.8	33.2	Type 2 diabetes
	CON		13	69	60.2	n/a	85.5	33.3	
Vasconcellos et al. (56)	SSG	RCT	6	33.33	13.9	160.9	85.1	30.5	Obesity/metabolic syndrome
	CON		7	28.57	14.7	163.1	84.7	30.8	
Mohammed et al. (57)^a^	SSG	RCT	10	100	17.8	156.0	58.2	23.7	Type 1 diabetes
	SDG		10	100	14.5	166.0	55.6	19.9	
	DG		10	100	15.6	157.0	53.2	21.7	
	CON		10	100	14.4	161.0	61.1	23.5	
Uth et al. (46)	SSG	RCT	33	n/a	47.4	n/a	72.6	25.5	Breast cancer
	CON		16	n/a	50.0	n/a	76.8	26.4	
McEwan et al. (55)	SSG	CT	9	100	56	n/a	100.6	33.4	Hypertension
	CON		7	100	60.0	n/a	98.0	32.1	
Skoradal et al. (53)^b^	SSG	RCT	27	50.9	60.0	172.0	85.0	28.6	Prediabetes
	CON		23	50.9	62.0	170.0	89.9	30.9	
Skoradal et al. (54)^b^	SSG	RCT	27	50.9	60.0	172.0	85.0	28.6	Prediabetes
	CON		23	50.9	62.0	170.0	89.9	30.9	
Krustrup et al. (52)	SSG	RCT	19	0	45.0	165.0	75.1	n/a	Hypertension
	CON		12	0	45.0	166.0	78.9	n/a	
Vieira de Sousa et al. (45)	SSG	RCT	22	43.1	61.0	n/a	n/a	33.0	Type 2 diabetes
	CON		29	43.1	61.0	n/a	n/a	32.7	
Uth et al. (50)^c^	SSG	RCT	29	100	67.1	177.0	83.4	26.6	Prostate cancer
	CON		28	100	66.5	180.8	89.0	27.6	
Uth et al. (51)^c^	SSG	RCT	29	100	67.1	177.0	83.4	26.6	Prostate cancer
	CON		28	100	66.5	180.8	89.0	27.6	
Sousa et al. (62)	SSG	RCT	19	52.63	58	n/a	88.9	32.7	Type 2 diabetes
	CON		15	33.33	58	n/a	82.9	33.1	
Mohr et al. (49)^d^	SSG	RCT	21	0	45.0	165.0	79.8	n/a	Hypertension
	CON		20	0	45.0	166.0	76.4	n/a	
Uth et al. (47)^c^	SSG	RCT	26	100	67.1	177.0	83.4	26.6	Prostate cancer
	CON		23	100	66.5	180.8	89.0	27.6	
Mohr et al. (19)^d^	SSG	RCT	21	0	45.0	165.0	79.8	n/a	Hypertension
	CON		20	0	45.0	166.0	76.4	n/a	
Andersen et al. (43)	SSG	CT	12	100	50.6	n/a	n/a	30.4	Type 2 diabetes
	CON		9	100	48.7	n/a	n/a	30.4	
Krustrup et al. (21)	SSG	RCT	22	100	46.0	n/a	97.8	30.0	Hypertension
	CON		11	100	46.0	n/a	97.8	30.0	
Knoepfli-Lenzin et al. (48)	SSG	RCT	15	100	37.0	n/a	n/a	26.0	Hypertension
	CON		17	100	38.0	n/a	n/a	27.0	
Andersen et al. (44)	SSG	RCT	13	100	46.7	181.0	100.1	30.4	Hypertension
	CON		9	100	47.8	182.0	100.0	30.0	

Reports sharing the same superscript letter originated from the same underlying trial or participant cohort. These linked reports were retained when they provided complementary outcome data and should not be interpreted as independent participant samples; N, analysis sample; Age, mean age of participants (years); BMI, body mass index (kg/m^2^); SSG, recreational football; CON, control; N/A, not reported.

**Table 2:** Intervention characteristics.

Author	Intervention	Fre	Intensity	Sets	Duration per set (min)	Intermittent recovery (min)	Total (min)	Number of competitors
Teixeira et al. (61)	3 months	2	81%HR_max_	4	10	2	40	3v3-6v6
Poffé et al. (59)	10 weeks	2	81%HR_max_	2	15	3	30	4v4-5v5
Mohammed et al. (60)^a^	12 weeks	2	n/a	2	45	n/a	90	3v3-5v5
Soares et al. (58)	12 weeks	3	n/a	2	12	3	24	3v3-7v7
Vasconcellos et al. (56)	12 weeks	3	85%HR_max_	1	40	n/a	40	2v2-4v4
Mohammed et al. (57)^a^	12 weeks	2	n/a	2	45	n/a	90	3v3-5v5
Uth et al. (46)	12 months	2	85%HR_max_	4	7	2	28	4v4-5v5
McEwan et al. (55)	8 weeks	1	n/a	n/a	n/a	n/a	60	n/a
Skoradal et al. (53)^b^	16 weeks	2	85%HR_max_	2	30	3	60	4v4-6v6
Skoradal et al. (54)^b^	16 weeks	2	85%HR_max_	2	30	3	60	4v4-6v6
Krustrup et al. (52)	12 months	3	n/a	4	12	n/a	48	4v4-8v8
Vieira de Sousa et al. (45)	12 weeks	3	75%HR_max_	2	12	3	24	3v3-7v7
Uth et al. (50)^c^	12 weeks	3	n/a	3	15	n/a	45	3v3-7v7
Uth et al. (51)^c^	32 weeks	3	n/a	3	15	n/a	45	3v3-7v7
Sousa et al. (62)	12 weeks	3	75%HR_max_	2	12	2	24	3v3-7v7
Mohr et al. (49)^d^	15 weeks	3	n/a	n/a	n/a	n/a	60	4v4-10v10
Uth et al. (47)^c^	12 weeks	3	85%HR_max_	3	15	n/a	45	5v5-7v7
Mohr et al. (19)^d^	15 weeks	3	89%HR_max_	n/a	n/a	n/a	n/a	4v4-10v10
Andersen et al. (43)	24 weeks	2	83%HR_max_	5	10	2	50	4v4-6v6
Krustrup et al. (21)	6 months	2	85% HR_max_	4	12	2	48	5v5-7v7
Knoepfli-Lenzin et al. (48)	12 weeks	3	80% HR_max_	n/a	n/a	n/a	50	4v4-10v10
Andersen et al. (44)	6 months	2	93% HR_max_	2	25	5	50	5v5-7v7

Reports sharing the same superscript letter originated from the same underlying trial or participant cohort. These linked reports were retained when they provided complementary outcome data and should not be interpreted as independent participant samples; N/A, not reported; Fre, training frequency (sessions per week); HR_max_, maximal heart rate.

### 3.3 Effects of recreational football on body composition

Recreational football produced a significant reduction in body fat percentage compared with control (13 trials; *k* = 13; SMD = −0.36, 95% CI − 0.57 to −0.14; *p* < 0.01; *I*^2^ = 3.22%; 95% PI −0.61 to −0.10; power = 16.5%; Egger’s *p* = 0.22). A small increase in muscle mass was indicated (11 trials; *k* = 12; SMD = 0.22, 95% CI 0.01 to 0.44; *p* = 0.05; *I*^2^ = 0%; power = 10.0%; Egger’s *p* = 0.93). The reduction in fat mass was borderline and was not retained under cluster-robust inference (12 trials; *k* = 13; SMD = −0.30, 95% CI − 0.59 to 0.00; *p* = 0.05). No statistically significant between-group effects were observed for body weight (13 trials; *k* = 13; SMD = −0.29, 95% CI − 0.58 to 0.00; *p* = 0.05), BMI (8 trials; *k* = 9; SMD = −0.06, 95% CI − 0.38 to 0.27; *p* = 0.71), or waist circumference (WC; 5 trials; *k* = 5; SMD = −0.48, 95% CI − 1.31 to 0.35; *p* = 0.18). Figure 2 summarizes the pooled body-composition outcomes.

**Figure 2:**
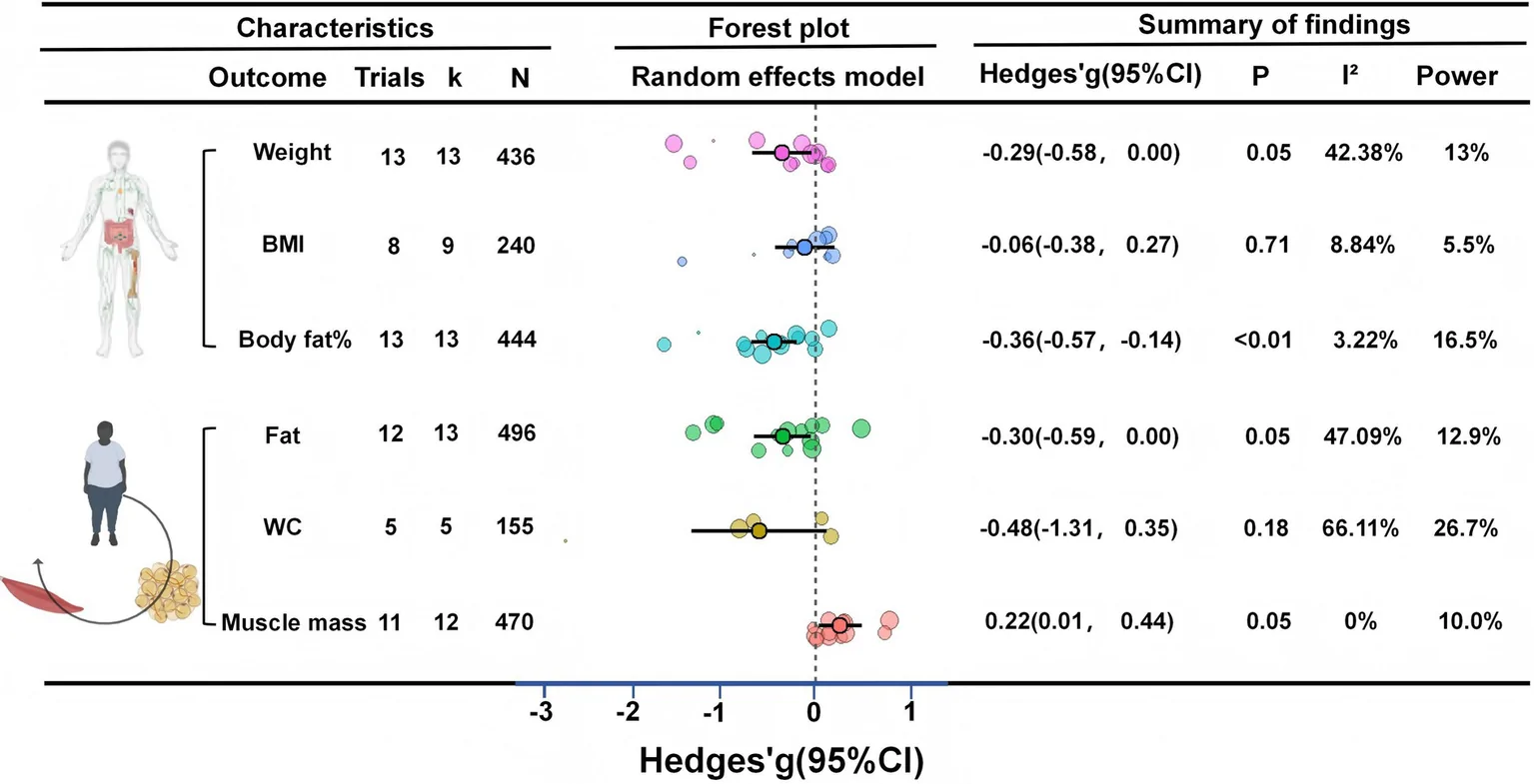
Orchard plot of the effects of recreational football on body composition. BMI, body mass index; WC, waist circumference; Fat, fat mass; g, Hedges’ g; Trials, the number of contributing studies in each mode; k, the total number of effects included in the pooled effect size; *I*^2^, heterogeneity; 95% CI, 95% confidence interval; Power, study-level statistical power.

### 3.4 Effects on cardiovascular and cardiorespiratory outcomes

Compared with control, recreational football was associated with significant reductions in diastolic blood pressure (DBP; 11 trials; *k* = 12; SMD = −0.53, 95% CI − 0.96 to −0.09; *p* = 0.02; *I*^2^ = 65.04%), mean arterial pressure (MAP; 7 trials; *k* = 7; SMD = −0.89, 95% CI − 1.48 to −0.29; *p* = 0.01; *I*^2^ = 67.85%), and resting heart rate (RHR; 9 trials; *k* = 10; SMD = −1.01, 95% CI − 1.72 to −0.31; *p* = 0.01; *I*^2^ = 83.11%; Egger’s *p* = 0.02). Cardiorespiratory fitness also improved significantly, with gains in VO₂peak (12 trials; *k* = 16; SMD = 0.70, 95% CI 0.37 to 1.03; *p* < 0.01; *I*^2^ = 60.03%) and Yo-Yo Intermittent Endurance Test level 1 performance (YYIE1; 4 trials; *k* = 4; SMD = 1.43, 95% CI 0.83 to 2.03; *p* < 0.01; *I*^2^ = 0%). The pooled effect for systolic blood pressure was borderline and was not retained under cluster-robust inference (SBP; 11 trials; *k* = 12; SMD = −0.60, 95% CI − 1.20 to 0.00; *p* = 0.05; *I*^2^ = 81.24%; Egger’s *p* = 0.01). Figure 3 summarizes these outcomes.

**Figure 3:**
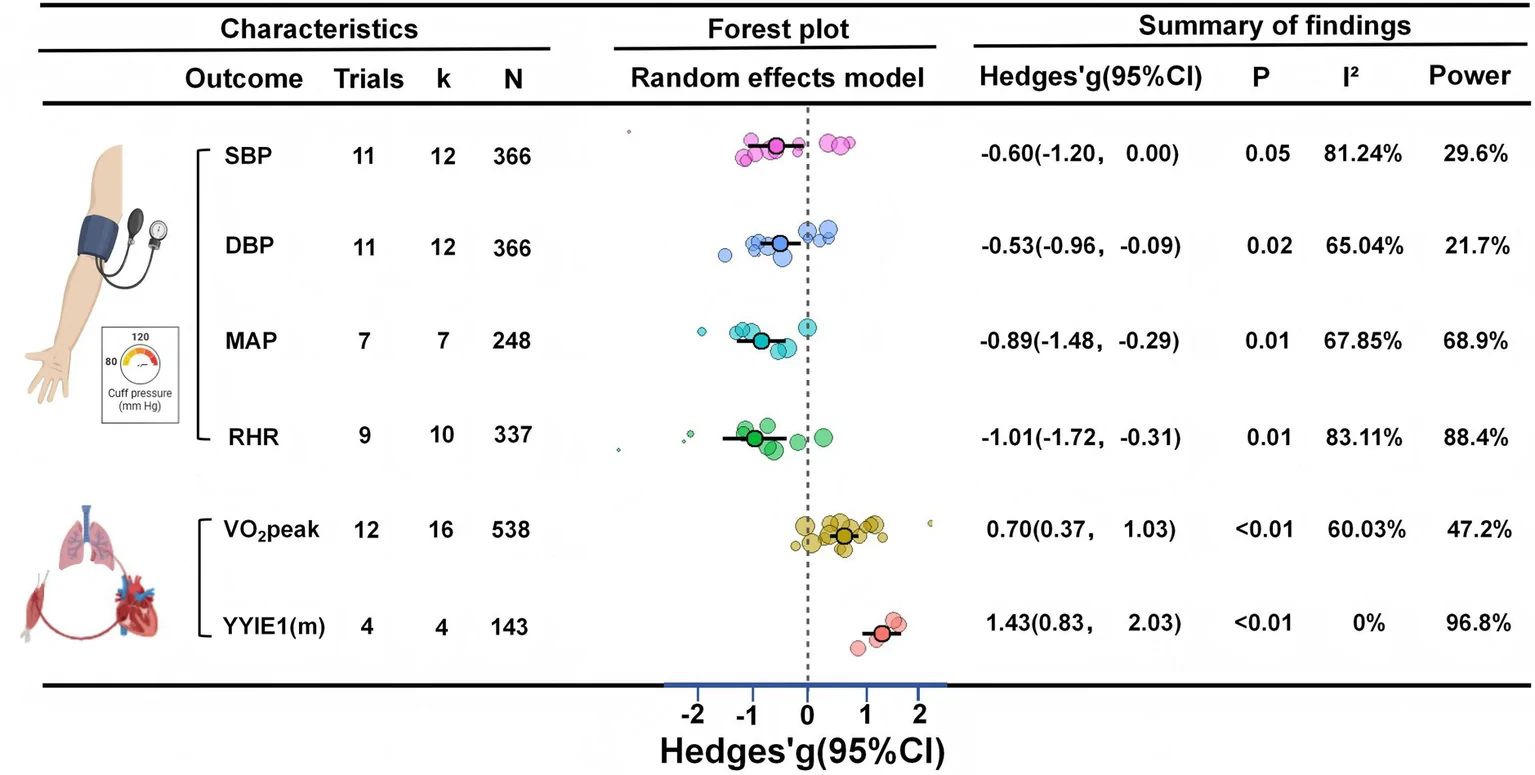
Orchard plot of the effects of recreational football on cardiovascular and cardiorespiratory outcomes. SBP, systolic blood pressure; DBP, diastolic blood pressure; MAP, mean arterial pressure; RHR, resting heart rate; YYIE1, Yo-Yo Intermittent Endurance Test level 1; *g*, Hedges’ *g*; Trials, the number of contributing studies in each mode; *k*, the total number of effects included in the pooled effect size; *I*^2^, heterogeneity; 95% CI, 95% confidence interval; Power, study-level statistical power.

### 3.5 Effects on lipid, inflammatory, and glycaemic outcomes

Recreational football was associated with significant reductions in triglycerides (10 trials; *k* = 11; SMD = −0.56, 95% CI − 0.91 to −0.22; *p* < 0.01; *I*^2^ = 40.72%) and total cholesterol (10 trials; *k* = 11; SMD = −0.69, 95% CI − 1.08 to −0.30; *p* < 0.01; *I*^2^ = 56.75%; Egger’s *p* = 0.04). The reduction in low-density lipoprotein (LDL; 10 trials; *k* = 11; SMD = −0.50, 95% CI − 1.01 to 0.00; *p* = 0.05) was borderline and was not retained under cluster-robust inference. No significant pooled effects were observed for HbA1c (6 trials; *k* = 7; SMD = −0.28; *p* = 0.10), fasting glucose (9 trials; *k* = 10; SMD = −0.11; *p* = 0.48), high-density lipoprotein (HDL; 11 trials; *k* = 12; SMD = 0.10; *p* = 0.70), C-reactive protein (CRP; 6 trials; *k* = 6; SMD = −0.88; *p* = 0.13; *I*^2^ = 89.36%), or fasting insulin (5 trials; *k* = 6; SMD = 0.07; *p* = 0.73). Figure 4 summarizes these outcomes.

**Figure 4:**
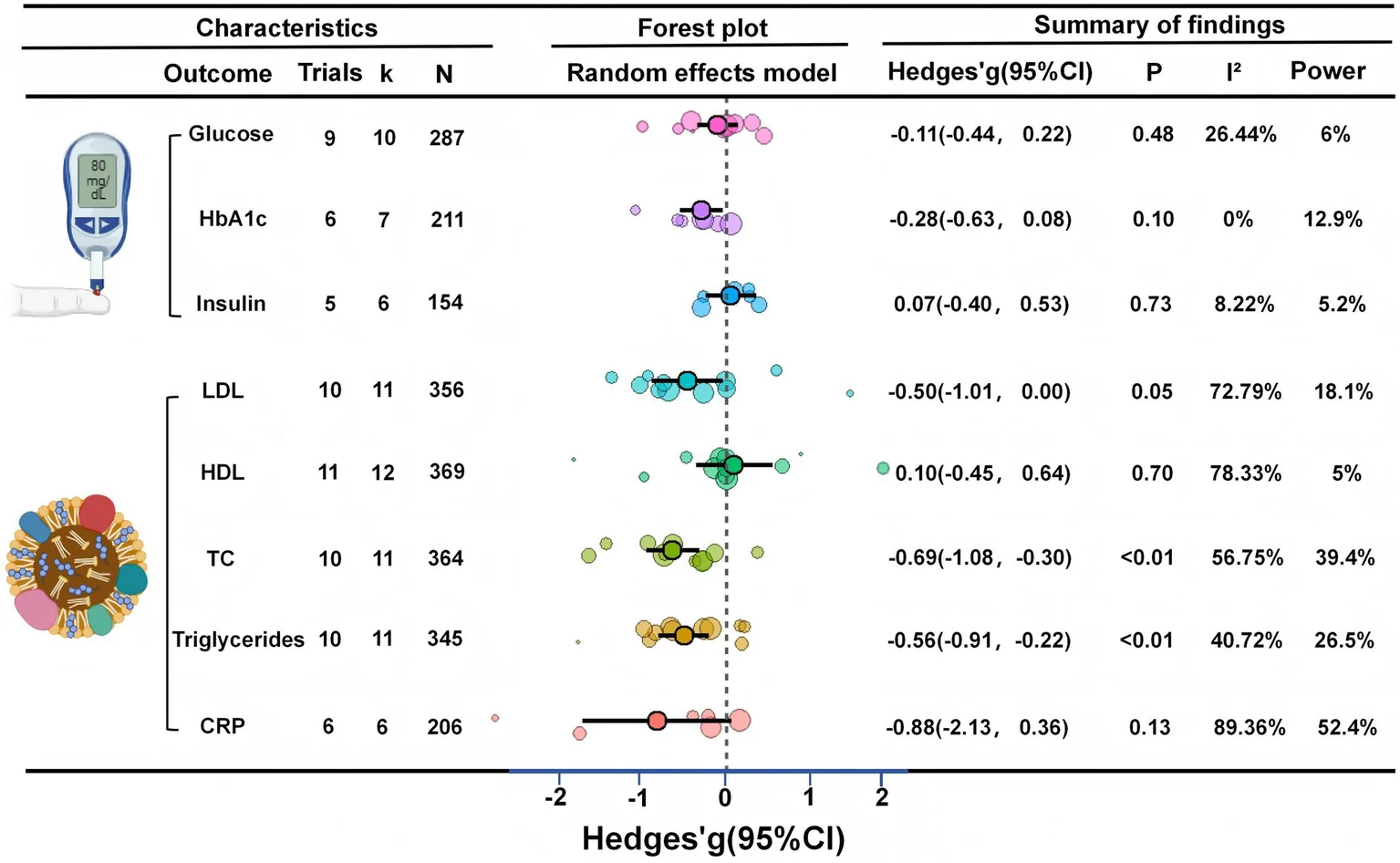
Orchard plot of the effects of recreational football on lipid, inflammatory, and glycaemic outcomes. TC, total cholesterol; CRP, C-reactive protein; *g*, Hedges’ *g*; Trials, the number of contributing studies in each mode; *k*, the total number of effects included in the pooled effect size; *I*^2^, heterogeneity; 95% CI, 95% confidence interval; Power, study-level statistical power.

### 3.6 Effects on bone health outcomes

No statistically significant effects were observed for leg BMD (3 trials; *k* = 3; SMD = 0.06; *p* = 0.77), whole-body BMD (7 trials; *k* = 7; SMD = 0.22; *p* = 0.11), leg BMC (3 trials; *k* = 3; SMD = 0.16; *p* = 0.48), whole-body BMC (6 trials; *k* = 6; SMD = 0.22; *p* = 0.14), or CTX-1 (3 trials; *k* = 4; SMD = 0.54; *p* = 0.19; *I*^2^ = 73.82%). Significant increases were observed for plasma osteocalcin (3 trials; *k* = 4; SMD = 1.07, 95% CI 0.53 to 1.60; *p* < 0.01; *I*^2^ = 0%) and P1NP (3 trials; *k* = 4; SMD = 1.28, 95% CI 0.22 to 2.34; *p* = 0.03; *I*^2^ = 71.84%), although the P1NP effect was not retained under cluster-robust inference (Supplementary Table S6). Figure 5 summarizes these outcomes.

**Figure 5:**
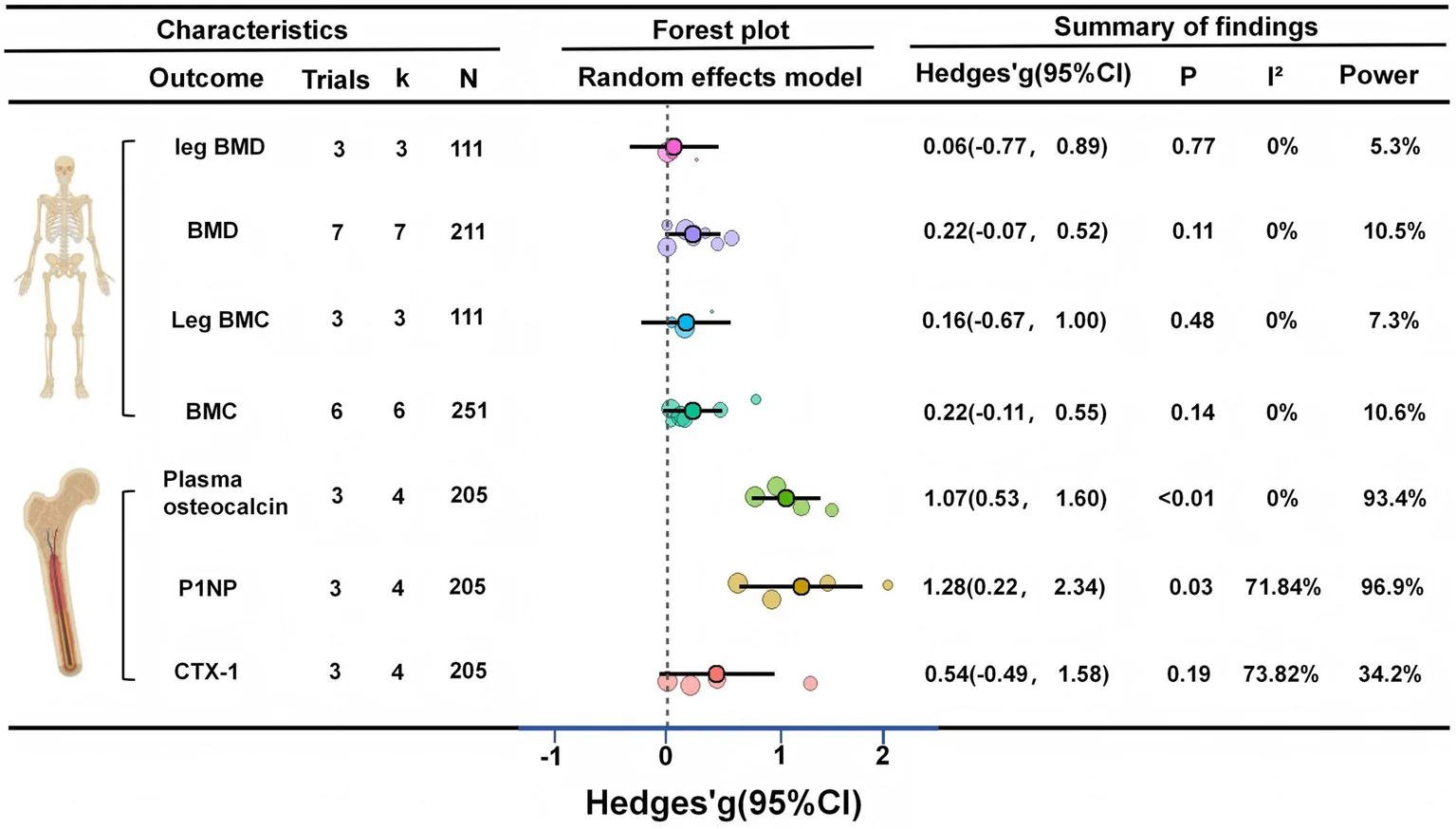
Orchard plot of the effects of recreational football on bone outcomes. BMD, bone mineral density; BMC, bone mineral content; P1NP, procollagen type I N-terminal propeptide; CTX-1, C-terminal telopeptide of type I collagen; *g*, Hedges’ *g*; Trials, the number of contributing studies in each mode; *k*, the total number of effects included in the pooled effect size; *I*^2^, heterogeneity; 95% CI, 95% confidence interval; Power, study-level statistical power.

### 3.7 Effects expressed as mean differences

In addition, to improve the clinical interpretability of the findings, mean differences (MDs) were calculated for outcomes reported on a consistent and clinically interpretable scale. Significant improvements were observed for body weight (13 trials; *k* = 13; MD = −1.46, 95% CI: −2.81 to −0.11; *I*^2^ = 45.94%; 95% PI: −4.60 to 1.67; power = 13.0%; *p* = 0.03), body fat percentage (13 trials; *k* = 13; MD = −0.87, 95% CI: −1.26 to −0.48; *I*^2^ = 0%; 95% PI: −1.26 to −0.47; power = 10.2%; *p* < 0.01), muscle mass (11 trials; *k* = 12; MD = 0.86, 95% CI: 0.22 to 1.50; *I*^2^ = 0%; 95% PI: 0.22 to 1.50; power = 9.7%; *p* = 0.01), MAP (7 trials; *k* = 7; MD = −5.10, 95% CI: −7.87 to −2.33; *I*^2^ = 59.24%; 95% PI: −11.26 to 1.05; power = 65.5%; *p* < 0.01), and RHR (9 trials; *k* = 10; MD = −5.73, 95% CI: −9.66 to −1.80; *I*^2^ = 88.24%; 95% PI: −17.45 to 5.98; power = 93.0%; *p* < 0.01). In contrast, no significant changes were observed for BMI (8 trials; *k* = 9; MD = −0.33, 95% CI: −1.22 to 0.57; *I*^2^ = 23.67%; 95% PI: −1.82 to 1.17; power = 5.5%; *p* = 0.42) or WC (5 trials; *k* = 5; MD = −3.83, 95% CI: −11.81 to 4.14; *I*^2^ = 90.53%; 95% PI: −22.28 to 14.60; power = 51.0%; *p* = 0.25).

### 3.8 Feasibility, adherence, and safety

Feasibility-related outcomes were inconsistently reported across the included studies. Attendance data were available from 13 independent trials, with a mean attendance rate of 82.7% (range, 47.5–100%), while attrition data were available from 14 independent trials, with a mean attrition rate of 15.6% (range, 0–32%). Adherence was described using achieved weekly training frequency, the number or proportion of training sessions attended or completed, and attainment of study-specific compliance thresholds. Information on intervention completion included completion of follow-up assessments, failure to initiate training, discontinuation of training, and withdrawal or exclusion from the study. Reported reasons for non-completion included personal or logistical constraints, low attendance, health conditions, and injuries. Where reported, training was supervised by coaches, investigators, exercise physiologists, physiotherapists, or physical trainers. Reported adverse events mainly involved the musculoskeletal system, including muscle soreness, strains, joint pain, and lower-limb injuries; serious injuries included fibular fractures and Achilles tendon injuries. Because definitions, denominators, and reporting approaches differed substantially across studies, feasibility and safety outcomes were summarized narratively rather than quantitatively pooled. Detailed study-level information is presented in Table 3.

**Table 3:** Feasibility and safety outcomes of the included studies.

Author	Adherence	Attendance	Attrition	Adverse events	Supervision	Intervention completion
Teixeira et al. (61)	The training frequency is 1.8 ± 0.7 times per week.	60.00%	2.50%	Ten participants experienced a total of 18 untimely injuries during the recreational five-a-side football intervention, which equates to 39.7 injuries per 1,000 h of training.	All sessions were conducted by a Physical Exercise and Health master degree student and supervised by the research team.	N/A
Poffé et al. (59)	11 participants achieved high compliance (≥80%).	83.70%	17.50%	Minor adverse events during the intervention included muscle soreness (n = 6), mild muscle strains in the calf, quadriceps, or hamstrings (n = 4), knee pain (n = 4), hip pain (n = 2), and rib contusion (n = 1). In the SSG group, one female participant and one male participant withdrew after the first training session due to personal reasons and recurrent Achilles tendinitis, respectively. The remaining three male participants withdrew due to calf muscle strains resulting from training (weeks 1, 3, and 8, respectively).	All training sessions were supervised by qualified coaches and were performed on an artificial football pitch.	In total, 5 participants (25%) of SSG and 2 (10%) of CON discontinued participation.
Mohammed et al. (60)	N/A	93.75%	0.00%	N/A	N/A	N/A
Soares et al. (58)	N/A	100.00%	0.00%	N/A	The soccer or running training protocol was supervised by a physical trainer.	N/A
Vasconcellos et al. (56)	100% of training bouts completed.	100.00%	N/A	N/A	Two licensed instructors ministered the soccer training, under the supervision of the principal investigator.	Six adolescents in each group dropped off before the beginning of the RSP due to financial reasons (albeit the program was free of charge, some families could not afford the transportation to the University facilities).
Mohammed et al. (57)	Participants in the football groups attended 21–24 sessions.	N/A	0.00%	N/A	N/A	N/A
Uth et al. (46)	0.8 ± 0.4 sessions/week among 12-month completers (n = 33).	47.46%	27.50%	During more than a year of football training, 15 participants sustained a total of 21 injuries. Most injuries were related to football training (n = 6) or matches (n = 6), and the majority involved the ankle, knee, or thigh muscles, resulting in a median absence of 2 weeks. One participant suffered a meniscus tear after a fall during indoor warm-up and did not return to football training.	The training was instructed and supervised by exercise physiologists or physiotherapists who also recorded attendance at each training session.	In total, 68 women were randomized to either the Football Fitness group (n = 46) or the control group (n = 22) after baseline assessment. Five participants allocated to FFG did not start the training intervention. One participant broke her leg while hiking on vacation after the baseline test and before the intervention started, and 4 regretted signing up to the trial for personal reasons, e.g., family and work obligations.
McEwan et al. (55)	Full attendance: 4/9 participants (44.4%) among those with post-intervention measurements.	90.00%	32.00%	N/A	N/A	In total, the recruitment strategy generated interest from 55 males of which 30 did not participate due to various extenuating circumstances. Of the 25 individuals (intervention: n = 12; control: n = 13) who provided informed consent and completed baseline measurements, 16 (intervention: n = 9; control: n = 7) completed measurements at the 8-week follow up. Reasons for drop out included family emergencies (n = 2), work commitments (n = 2), and personal health complications unrelated to the study (n = 2). No subsequent contact was made by the remaining participants (n = 2) as to explain their reasons for dropping out. A relatively high retention rate of 68% was therefore achieved in the present study.
Skoradal et al. (53)	N/A	N/A	N/A	There were examples of severe muscle soreness as result of the football training in the initial phase of the intervention period, but no serious injuries occurred.	N/A	There were five dropouts from the SSG group (due to low training attendance; <1 session per week, which was the minimum criteria for training completion), of which two were men and three were women, resulting in 27 participants completing the SSG intervention. There were no dropouts from CON (n = 23). Thus, the final sample consisted of 50 participants.
Skoradal et al.(54)	The football training group completed a total of 32 ± 2 (range: 19–46) football training sessions over the 16-week intervention period, corresponding to 2.0 ± 0.1 (1.2–2.9) weekly sessions.	N/A	15.63%	N/A	N/A	N/A
Krustrup et al. (52)	2.5 ± 0.4 sessions/week (range, 1.8–3.5).	83.33%	24.39%	One SSG participant suffered an Achilles tendon rupture during the first week of training.	A trained soccer coach was present during all training sessions to control the duration of the training, ensure competitive games, and record training attendance.	One participant in SOC suffered an Achilles tendon rupture during the first week of training, while two other participants were excluded from the study due to relocation (n = 1) and low training attendance (n = 1; <1.5 sessions per week). A total of 12 participants from the control group participated in the post-testing, whereas eight participants did not due to relocation (n = 2), pregnancy (n = 1), initiation of physical training (n = 4), and disease (n = 1).
Vieira de Sousa et al. (45)	N/A	N/A	19.61%	N/A	N/A	Five subjects dropped out of the study (SSG, n = 1; CON, n = 4). Five subjects were excluded due to: cardiovascular disease and hyperparathyroidism (n = 1); diabetic foot (n = 1); started on insulin therapy (n = 1); and could not continue with the training schedule (n = 2).
Uth et al. (50)	SSG participants who completed both the baseline and 12-week assessments attended a mean of 20.6 ± 8.0 (±SD) of the 28 training sessions.	76.50%	N/A	Five SSG participants suffered musculoskeletal injuries in separate incidents unrelated to tackles or other contact with teammates. Two fibular fractures and one partial Achilles tendon rupture were classified as serious, according to guidelines. All five injured participants recovered after conservative treatment, and three resumed football training during the study period. One participant withdrew from the study and was not included in follow-up, and another was excluded from training due to peripheral neuropathy discovered during post-injury treatment.	An experienced instructor supervised all training sessions.	N/A
Uth et al. (51)	Weeks 0–12: 1.6 ± 0.5 sessions/week (n = 26); weeks 12–32: 0.9 ± 0.5 sessions/week (n = 21).	56.30%	28.07%	In SSG, two participants sustained a fibula fracture and three participants had muscle or tendon injuries due to the football training. All injuries occurred within the first 12 weeks. After conservative treatment, all injured participants regained normal musculoskeletal function. After 6–8 weeks of recovery, three resumed football training in the study period. The three participants that resumed training after recovery played football for a total of 24–26 weeks.	An experienced exercise physiologist or physiotherapist supervised all training sessions.	One participant withdrew from the study and was not included in subsequent follow-up, while another was excluded from training after being diagnosed with peripheral neuropathy during recovery from an injury.
Sousa et al. (62)	N/A	N/A	22.73%	N/A	N/A	Five subjects dropped out of the study (SSG, n = 1; CON, n = 4; 4 did not attend and 1 had a bicycle accident). Another five were excluded due to cardiovascular disease and hyperparathyroidism (n = 1); diabetic foot (n = 1); started on insulin therapy (n = 1); and could not continue with the training schedule (n = 2). A total of 34 participants completed the study, 19 of whom were in SSG (9 females and 10 males) and 15 were in CON (10 females and 5 males).
Mohr et al. (49)	3.0 ± 0.5 sessions/week (range, 2.7–3.5).	N/A	N/A	N/A	An experienced soccer coach was present during all training sessions to control the duration of the training and ensure competitive game play.	N/A
Uth et al. (47)	Participants in FG who completed pre- and post-intervention assessments attended a mean of 20.6 (SD 8.0) of the 28 training sessions, corresponding to an attendance rate of 76.5% (SD 24.2).	76.50%	14.04%	In SSG, two participants had a fracture of the fibula bone (session 1 and 16, respectively). Also, one participant had a partial rupture of an Achilles tendon (session 3), and one participant had an ankle sprain in training session 16. All four recovered fully from these injuries after conservative treatment. Additionally, one participant withdrew from the study after a quadriceps muscle strain. In one of the participants who had a fibula fracture, a diagnosis of peripheral neuropathy was revealed in the medical treatment following the fracture and the participant was therefore excluded from the intervention but re-tested at 12 weeks and the data were included in the analysis. Three of the injured participants resumed football training after appropriate recovery time.	All training sessions were supervised by an experienced instructor.	N/A
Mohr et al. (19)	N/A	100.00%	N/A	One additional subject in the SSG suffered an Achilles tendon rupture during the first week of training and was omitted from the group. Beside this incident, no major injuries occurred. Some subjects had to skip certain training sessions due to muscle soreness, but no one was absent for more than three consecutive sessions.	A trained football coach was present during all training sessions in order to control the duration of the training and to ensure competitive games.	N/A
Andersen et al. (43)	The average total number of training sessions during IP was 37.6 (1.5 sessions per week). The average weekly training attendance was the same during the initial 12 weeks and the last 12 weeks of the intervention period (1.56 ± 0.09 (± SEM) vs. 1.47 ± 0.10 sessions per week).	75.00%	N/A	N/A	Intervention under coach supervision	N/A
Krustrup et al. (21)	The total number of training sessions was 43 ± 13 (1.7 ± 0.5 sessions/week).	N/A	9.10%	Two participants in the SSG group withdrew due to injury during study-related training.	Each training session lasted 1 h, and sessions were supervised by one of the investigators.	During months 3–6, six SSG participants and four CON participants withdrew; two SSG withdrawals were due to study-related injuries, and the remaining withdrawals were due to inability to adhere to the study protocol.
Knoepfli-Lenzin et al. (48)	Football group: 2.4 ± 0.2 sessions/week.	80.00%	13.95%	Two football-group participants withdrew because of study-related injuries.	All training sessions were supervised by an investigator with the help of on-line heart rate monitoring in order to ensure that the heart rate of the participants was above the 65% maximal heart rate.	Four participants (F: 1, C: 3) withdrew from the study because of lack of time, while two participants withdrew because of injuries (F: 2, related to the study).
Andersen et al. (44)	The total number of training sessions was 22 ± 2 (1.7 ± 0.2/week).	85.00%	12.00%	One participant each from the SSG and CON groups withdrew due to illness. Another participant from the SSG group withdrew due to a knee injury sustained during study-related soccer training.	Each training session lasted 1 h and sessions were supervised by one of the investigators.	N/A

Reports sharing the same superscript letter originated from the same underlying trial or participant cohort. These linked reports were retained when they provided complementary outcome data and should not be interpreted as independent participant samples; N/A, not reported; HR_max_, maximal heart rate.

### 3.9 Sensitivity analyses

Sensitivity analyses using the leave-one-out method, performed at the level of the independent trial, showed that the robustness of the pooled findings varied across outcomes. Significant effects for body fat percentage, DBP, MAP, RHR, VO₂peak, YYIE1 performance, total cholesterol, triglycerides, plasma osteocalcin, and P1NP were retained after the sequential exclusion of individual trials. Similarly, the overall non-significant findings for BMI, WC, glucose, insulin, HDL, leg bone mineral density, leg bone mineral content, and whole-body bone mineral content remained unchanged. In contrast, the statistical significance of the pooled effects for weight, fat mass, muscle mass, SBP, LDL, HbA1c, CRP, whole-body BMD and CTX-1 varied following the exclusion of individual trials, indicating that these findings were sensitive to the influence of particular trials. Overall, the direction of the pooled effects remained largely consistent across analyses, but several outcomes should be interpreted with caution because of limited robustness. Detailed results are provided in Supplementary Figures S2–S5.

Sensitivity analyses using alternative pre–post correlation coefficients (*r* = 0.20, 0.50, and 0.80) generally supported the findings obtained using the primary assumption of *r* = 0.85. For most outcomes, the direction and statistical interpretation of the pooled effects remained consistent across the alternative correlation assumptions. However, the findings for body fat percentage, fat mass, muscle mass, SBP, and LDL were sensitive to the assumed correlation coefficient and were therefore considered non-robust. Weight remained consistently borderline across all correlation assumptions. Although the direction and statistical interpretation remained unchanged for DBP, MAP, VO₂peak, RHR, YYIE1 performance, triglycerides, total cholesterol, plasma osteocalcin, and P1NP, the magnitude of the pooled effects varied across correlation assumptions, with generally larger estimates observed as the assumed correlation increased. Overall, the primary conclusions were largely robust to alternative pre–post correlation assumptions, although the magnitude or statistical inference of several outcomes showed some sensitivity to the assumed correlation coefficient. Detailed results are provided in Supplementary Table S5.

After excluding non-randomized controlled trials, the overall findings remained generally robust. This analysis was restricted to weight, BMI, and VO₂peak, as these were the only outcomes with two contributing non-randomized controlled trials; other outcomes included only one or no such trial and were therefore not examined (Supplementary Table S10). The results for BMI and VO₂peak were unchanged, while the finding for weight was directionally consistent with the primary analysis but remained borderline significant. Detailed results are provided in Supplementary Table S4.

Cluster-robust (CR2) inference, clustering on the independent trial, was applied to all 27 outcome models (Supplementary Table S6) and to the mean-difference models (Supplementary Table S7). The number of contributing trials ranged from 3 to 13 and the Satterthwaite degrees of freedom from 1.7 to 11.4, falling below four in 8 of the 27 models. Pooled estimates were unchanged and the statistical interpretation was concordant for 22 of the 27 outcomes. The borderline effects on fat mass, SBP and LDL (all *p* = 0.05 under model-based inference) and the increase in P1NP (95% CI − 0.17 to 2.74; *p* = 0.06; df = 2.0) were not retained under cluster-robust inference, whereas whole-body BMD became nominally significant (95% CI 0.03 to 0.42; *p* = 0.03; df = 5.6); among the mean differences, the reduction in body weight was likewise not retained (*p* = 0.05; df = 7.3). For these six outcomes the two inferential procedures disagree, and no conclusion is drawn from them. Varying the assumed within-trial sampling correlation across ρ = 0.20 to 0.80 changed no pooled estimate by more than 0.03; the only conclusion affected was muscle mass, whose model-based *p* value moved from 0.04 at ρ = 0.20 to 0.05 at ρ = 0.80 while remaining significant under cluster-robust inference throughout (Supplementary Table S8).

### 3.10 Risk of bias and certainty of evidence

Among the 15 independent randomized controlled trials assessed using RoB 2, 73.3% (11/15) were judged as having some concerns in the randomization process, 86.7% (13/15) in the missing outcome data domain, 80.0% (12/15) in measurement of the outcome, and 73.3% (11/15) in selection of the reported result. All 15 randomized trials were judged at low risk for deviations from intended interventions, while all were judged as having some concerns overall. The two non-randomized controlled studies were assessed separately using ROBINS-I; both were judged to have moderate risk of bias overall, with moderate risk in the confounding, participant-selection, deviations-from-intended-interventions, missing-data, and selective-reporting domains, and low risk in classification of interventions and measurement of outcomes.

Across outcomes, certainty of evidence ranged from low to very low: low-certainty evidence was rated for body fat percentage, Yo-Yo Intermittent Endurance Test performance, plasma osteocalcin, and P1NP, while most remaining outcomes were rated as very low certainty. Detailed risk-of-bias assessments are provided in Supplementary Figure S1, and outcome-level GRADE assessments are provided in Supplementary Table S2.

## 4 Discussion

### 4.1 Principal findings

Across the selected chronic disease populations represented in the available evidence, recreational football showed statistically significant pooled effects on body fat percentage, muscle mass, DBP, MAP, RHR, VO₂peak, Yo-Yo intermittent endurance performance, triglycerides, total cholesterol, and plasma osteocalcin. The model-based analysis also indicated an increase in P1NP; however, this finding was not retained under cluster-robust (CR2) inference and was therefore not considered robust. Borderline effects on fat mass, SBP, and LDL were likewise not retained under cluster-robust inference. Pooled effects on body weight, BMI, WC, HbA1c, fasting glucose, HDL, CRP, DXA-derived bone mineral density and content, and CTX-1 were not statistically significant. However, sensitivity analyses across pre–post correlation assumptions ranging from *r* = 0.20 to 0.85 showed that the statistical significance of body fat percentage, fat mass, muscle mass, SBP, and LDL was assumption-sensitive. These five findings should be regarded as non-robust and interpreted more cautiously than outcomes whose direction and statistical interpretation were consistent across all correlation assumptions. More broadly, the pooled estimates should not be read as evidence of uniform benefit across all chronic disease populations, nor as evidence that recreational football can substitute for established exercise prescriptions (4, 7, 9).

### 4.2 Body composition

The pattern of reduced body fat percentage without consistent changes in body weight, BMI, fat mass, or WC is consistent with earlier football interventions in untrained adults, in which reductions in adiposity have been more reproducible than changes in scale weight (13, 24, 63, 64). A mechanistic contribution from concurrent lean-tissue preservation or gain is plausible: Uth et al. (47) reported a between-group difference in lean body mass change of approximately 0.7 kg after 12 weeks, favouring the football group. Scale-based indices (body weight, BMI) and circumference measures are known to be relatively insensitive to compositional change over 8 to 52 weeks, particularly when interventions are not paired with sustained energy restriction and when participants are receiving pharmacological therapy with weight-active effects (4, 20, 62).

### 4.3 Cardiovascular and cardiorespiratory outcomes

The most consistent signal in the present synthesis is the improvement in cardiorespiratory fitness, with SMDs of 0.70 for VO₂peak and 1.43 for Yo-Yo intermittent endurance. The direction and magnitude align with the dedicated VO₂max meta-analysis by Milanović et al. (17), in which recreational football produced a mean increase of approximately 3.5 mL·kg^−1^·min^−1^ across mixed adult cohorts, and with the broader physical-fitness meta-analysis by the same group (64). However, the VO₂peak prediction interval crossed zero and certainty was very low, indicating that the average benefit was not reproduced consistently across diagnoses and programmes. That variability is expected given the clinical span of included trials, including hypertension, type 2 diabetes, breast and prostate cancer (including androgen-deprivation-treated patients), and adolescent metabolic-risk cohorts, which differ substantially in baseline physiology, treatment burden, and exercise tolerance (64).

The reductions in DBP (SMD − 0.53), MAP (SMD − 0.89), and RHR (SMD − 1.01) are directionally coherent with small-sided football trials in hypertensive adults, in which 3-month training reduced DBP by approximately 8 mmHg and RHR by approximately 5 to 8 bpm (19, 44, 64). The borderline pooled effect for SBP, which was not retained under cluster-robust inference, is at first sight inconsistent with those same trials, but is readily explained by high between-study heterogeneity, imprecision, possible small-study effects (Egger’s *p* = 0.01), and the inclusion of treated hypertensive cohorts in whom medication may have reduced residual headroom for further pressure lowering (13). Together, these haemodynamic results are compatible with adaptations plausibly mediated by improved stroke volume, peripheral oxygen extraction, endothelial function, and autonomic modulation, all previously described after football training (12, 13, 24). They do not, however, support the assumption that recreational football reliably lowers systolic pressure across all chronic disease populations.

### 4.4 Lipid and glycaemic outcomes

The significant reductions in triglycerides (SMD − 0.56) and total cholesterol (SMD − 0.69) are in line with prior football trials in sedentary adults and in type 2 diabetes, where triglyceride lowering has been among the more reproducible metabolic responses (13, 45, 62, 64). Certainty remained very low and the prediction intervals included zero for both outcomes, so the pooled means should not be interpreted as stable, generalizable effects. The absence of a pooled effect on HDL, and the borderline, non-robust estimate for LDL, are consistent with the recognized sensitivity of these fractions to energy balance, dietary composition, body-fat redistribution, medication, and intervention duration, all of which varied across included trials.

Glycaemic outcomes were largely unchanged at the pooled level, despite earlier diabetes-specific football trials reporting improved insulin sensitivity or reduced HbA1c at the individual-study level (43, 62). Plausible contributors to the pooled null include small sample sizes, short exposure, inclusion of participants whose glycaemia was already pharmacologically controlled, and the limited sensitivity of fasting indices and HbA1c to modest exercise effects in mixed clinical samples (9). The CRP result is best read as uninformative rather than negative, given the width of the interval and the small number of contributing studies.

### 4.5 Bone outcomes

The bone findings, namely, significant increases in plasma osteocalcin and, under model-based inference only, P1NP without detectable changes in DXA-derived density or content or in CTX-1, are compatible with an early anabolic response to mechanical loading that has not yet translated into measurable structural remodeling over the intervention periods studied. Bone turnover markers can respond within weeks to altered loading, whereas DXA-derived density and content measures typically require longer exposure and are more sensitive at mechanically loaded sites than to whole-body adaptations (18, 65). The same Sports Medicine meta-analysis reported larger and more consistent effects on leg BMD and BMC than on whole-body indices, and included studies in younger, longer-exposed cohorts with stronger lower-limb loading profiles (18). In the present chronic disease evidence base, older age, cancer treatment, lower tolerated training dose, and the frequent use of whole-body rather than region-specific outcomes would all be expected to attenuate structural change (47, 66). The osteocalcin estimate appears more robust than the P1NP estimate because its prediction interval remained on the beneficial side of the null, whereas the P1NP interval crossed zero with substantial heterogeneity. The evidence therefore supports osteogenic signalling but does not permit a confident claim of short- to medium-term improvement in clinically relevant bone density or content in chronic disease cohorts.

### 4.6 Mechanistic interpretation

The physiological profile of recreational football combining sustained cardiovascular load with brief high-intensity efforts, repeated accelerations and decelerations, turns, jumps, and ball contacts provides a coherent basis for the observed concurrent improvements in VO₂peak, RHR, body fat percentage, and bone formation markers (18, 24, 63, 67, 68). Outcomes that remained unchanged in the pooled analysis (BMI, WC, SBP, HbA1c, DXA indices) are plausibly those requiring a larger cumulative training dose, concurrent nutritional intervention, longer exposure, or more homogeneous patient samples than the current evidence base provides. Because few included trials were designed to isolate mediators, these mechanistic interpretations should be treated as hypotheses supported by the broader football physiology literature rather than as conclusions drawn from the present synthesis.

### 4.7 Disease-specific qualitative interpretation

Although the available evidence was insufficient for disease-specific meta-analysis, several qualitative patterns were observed. In hypertension, recreational football was generally associated with improvements in cardiorespiratory fitness, RHR, and selected haemodynamic outcomes, although blood-pressure effects were inconsistent, particularly in participants receiving antihypertensive treatment (19, 21, 44, 48, 49, 52, 55, 59, 61). Interpretation is limited by small samples, differences in medication status, and variation in intervention duration and football format.

In diabetes, studies involving adults with type 2 diabetes reported potential benefits for fitness (43, 45, 58, 62), body composition, lipid profile, and insulin resistance, whereas effects on HbA1c and fasting glucose were inconsistent. Evidence for type 1 diabetes was derived from two linked reports of the same small adolescent cohort receiving concurrent caloric control (57, 60), while two additional linked reports involved participants with prediabetes (53, 54). These factors limit conclusions regarding diabetes-specific efficacy.

In cancer (46, 47, 50, 51), one breast-cancer study and several linked reports from a single prostate-cancer trial suggested possible benefits for cardiovascular fitness, lean body mass, physical function, and selected bone-related outcomes. However, the small, highly selected, treatment-specific samples, variable attendance, and reported musculoskeletal injuries limit generalizability to other cancer populations. Overall, these disease-specific findings should be considered preliminary and hypothesis-generating rather than definitive evidence of efficacy.

### 4.8 Feasibility, adherence, and safety

The feasibility assessment suggests that recreational football may achieve generally acceptable participation and retention in selected supervised settings, with a mean attendance rate of 82.7% across 13 independent trials and a mean attrition rate of 15.6% across 14 independent trials reporting these outcomes. Adherence was described using achieved training frequency, session attendance or completion, and study-specific compliance thresholds, whereas intervention completion encompassed differing definitions of training discontinuation, study withdrawal, and follow-up completion. Supervision was reported in several studies, although its description varied. Substantial variation across studies indicates that feasibility is likely to depend on population characteristics, programme duration, scheduling, supervision, and clinical context. Moreover, most interventions were delivered in structured research settings, limiting conclusions regarding longer-term acceptability and implementation in routine clinical or community practice.

Safety requires similarly cautious interpretation. Adverse-event reporting was incomplete and heterogeneous across studies, with most reported events being musculoskeletal, including muscle soreness, strains, joint pain, and lower-limb injuries, although more serious injuries were reported in individual trials. Poffé et al. (59) reported predominantly mild-to-moderate musculoskeletal complaints and injuries in older adults, with several injury-related withdrawals, and noted that many injuries occurred during the earlier phase of training, highlighting the potential importance of progressive exposure and injury-prevention strategies. Teixeira et al. (61) also reported a relatively high injury incidence during recreational futsal training in men with treated hypertension, while noting that orthopaedic injuries also occurred outside the intervention, making attribution to football participation alone uncertain. Football-specific studies in oncology and diabetes populations indicate that supervised community- or clinic-linked delivery can be feasible; however, the infrequent reporting of serious harms should not be interpreted as evidence that such events were absent (45–47, 66). These findings suggest that the potential health benefits of recreational football should be balanced against its musculoskeletal demands, particularly in previously untrained, older, or clinically vulnerable participants. Pre-participation screening, qualified supervision, appropriate warm-up and familiarization, gradual progression of training load, and systematic injury monitoring may therefore be important components of programme delivery. Given the incomplete and inconsistent reporting of adverse events, firm conclusions regarding diagnosis-specific safety or scalability in routine clinical and community settings cannot yet be drawn.

### 4.9 Clinical interpretation

For appropriately selected patients who prefer group-based and game-based physical activity and who can participate safely after clinical screening, recreational football may be considered as a supervised adjunctive exercise option alongside established exercise and clinical care. The current evidence suggests potential benefits particularly for cardiorespiratory fitness and selected haemodynamic and metabolic outcomes; however, these findings should be interpreted cautiously given the substantial clinical heterogeneity across the included populations and the predominantly low or very low certainty of evidence. The limited number of studies within individual diagnostic categories also precludes firm disease-specific recommendations. Accordingly, recreational football should not be regarded as an established alternative to conventional exercise therapy or as a disease-specific therapeutic prescription. Its use should instead be individualized according to diagnosis, treatment and medication status, baseline functional capacity, deconditioning, musculoskeletal risk, and the availability of appropriate supervision (4, 9, 66).

### 4.10 Strengths and limitations

The main strength of this review is a quantitative synthesis restricted to selected chronic disease populations across multiple physiological domains, using three-level models with model-based and cluster-robust inference reported in parallel, and reporting prediction intervals alongside GRADE certainty. Principal limitations are the small number of studies per outcome, low power, substantial clinical and methodological heterogeneity, and very low certainty for most pooled estimates. With three to thirteen independent trials per outcome, the small-sample degrees of freedom of the cluster-robust estimator were also low, so both inferential procedures are reported, and substantive claims are restricted to the outcomes on which they agree. In particular, the included populations encompassed hypertension, type 1 and type 2 diabetes, cancer, and broader metabolic-risk profiles, which differ in underlying pathophysiology, treatment burden, baseline functional capacity, and potential responsiveness to exercise. Several statistically significant findings had prediction intervals spanning the null, indicating meaningful variability in true effects across settings. Accordingly, the pooled estimates should be interpreted as average effects across the populations represented in the current evidence base rather than as effects that are uniformly generalizable to all chronic diseases. The limited number of studies within individual diagnostic categories precluded robust diagnosis-specific conclusions. Accordingly, the pooled findings should be interpreted as overall signals across the selected chronic disease populations represented in the available evidence, rather than as evidence of efficacy for hypertension, diabetes, cancer, or any other single diagnosis. Adequately powered diagnosis-specific trials are required to determine whether the magnitude and clinical relevance of the effects differ across disease groups. These limitations do not negate the observed signals of benefit, but they materially constrain inferences about effect size.

## 5 Conclusion

Across selected chronic disease populations, recreational football showed signals of potential benefit across several physiological domains, particularly cardiorespiratory fitness. Certainty of evidence is predominantly low or very low, and several pooled effects show prediction intervals that include the null, so the present results should be read as context-dependent signals of potential benefit rather than evidence of definitive or uniformly generalizable efficacy across chronic disease populations. Recreational football may therefore be considered only as a supervised adjunctive exercise option for appropriately selected patients, rather than as an established alternative to conventional exercise therapy, particularly where cardiorespiratory fitness is the target. Adequately powered, diagnosis-specific trials with standardised reporting of training dose, adherence, and adverse events are required before firm clinical recommendations can be made.
