# Supplementary material for: Recreational football as medicine? A meta-analysis of its effects and feasibility in patients with chronic diseases
Source: Front Public Health. 2026 Oct 19;14:1869982. doi: 10.3389/fpubh.2026.1869982 (PMC13724509; doi:10.3389/fpubh.2026.1869982)
Supplement: Supplementary file 1 [file Data_Sheet_1.docx]

**Table S1 Search strategy**

|  | **Search terms** |
| --- | --- |
| **#1** | **Recreational soccer**  **((small-sided games) OR (recreational soccer)) OR (football fitness)** |
| **#2** | **Chronic Diseases**  **(diabetes) OR (hypertension) OR (Prostate cancer) OR (chronic)** |
| **#3** | **#1 AND #2** |

| **Table S2 GRADE** | | | | | | | | |
| --- | --- | --- | --- | --- | --- | --- | --- | --- |
| **Outcome** | **No. of effect sizes (No. of trials )** | **Certainty Assessment** | | | | | **Standardized Mean effect (95% CI)** | **GRADE*** |
|  |  | **Risk of Bias** | **Inconsistency** | **Indirectness** | **Imprecision** | **Other** |  |  |
| **Body Composition** | | | | | | | | |
| **Body Weight** | **13(13)** | **Serious** | **Serious** | **Serious** | **Serious** | **None** | **-0.29 (-0.58 to 0.00)** | ⨁◯◯◯ **VERY LOW** |
| **BMI** | **9(8)** | **Serious** | **Not serious** | **Serious** | **Serious** | **None** | **-0.06 (-0.38 to 0.27)** | ⨁◯◯◯ **VERY LOW** |
| **Body Fat Percentage** | **13(13)** | **Serious** | **Not serious** | **Serious** | **Serious** | **None** | **-0.36 (-0.57 to -0.14)** | ⨁⨁◯◯  **LOW** |
| **Fat Mass** | **13(12)** | **Serious** | **Serious** | **Serious** | **Serious** | **None** | **-0.30 (-0.59 to 0.00)** | ⨁◯◯◯ **VERY LOW** |
| **WC** | **5(5)** | **Serious** | **Serious** | **Serious** | **Serious** | **None** | **-0.48 (-1.31 to 0.35)** | ⨁◯◯◯ **VERY LOW** |
| **Muscle mass** | **12(11)** | **Serious** | **Not serious** | **Serious** | **Serious** | **None** | **0.22 (0.01 to 0.44)** | ⨁◯◯◯ **VERY LOW** |
| **Cardiometabolic health** | | | | | | | | |
| **SBP** | **12(11)** | **Serious** | **Serious** | **Serious** | **Serious** | **Publication Bias** | **-0.60 (-1.20 to 0.00)** | ⨁◯◯◯ **VERY LOW** |
| **DBP** | **12(11)** | **Serious** | **Serious** | **Serious** | **Serious** | **None** | **-0.53 (-0.96 to -0.09)** | ⨁◯◯◯ **VERY LOW** |
| **MAP** | **7(7)** | **Serious** | **Serious** | **Serious** | **Serious** | **None** | **-0.89 (-1.48 to -0.29)** | ⨁◯◯◯ **VERY LOW** |
| **Resting**  **Heart**  **Rate** | **10(9)** | **Serious** | **Serious** | **Serious** | **Not serious** | **Publication Bias** | **-1.01 (-1.72 to -0.31)** | ⨁◯◯◯ **VERY LOW** |
| **VO_2_peak** | **16(12)** | **Serious** | **Serious** | **Serious** | **Serious** | **None** | **0.70 (0.37 to 1.03)** | ⨁◯◯◯ **VERY LOW** |
| **YYIE1** | **4(4)** | **Serious** | **Not serious** | **Serious** | **Not serious** | **None** | **1.43 (0.83 to 2.03)** | ⨁⨁◯◯  **LOW** |
| **Fasting**  **Glucose** | **10(9)** | **Serious** | **Serious** | **Serious** | **Serious** | **None** | **-0.11 (-0.44 to 0.22)** | ⨁◯◯◯ **VERY LOW** |
| **HbA1c** | **7(6)** | **Serious** | **Not serious** | **Serious** | **Serious** | **None** | **-0.28 (-0.63 to 0.08)** | ⨁◯◯◯ **VERY LOW** |
| **Fasting**  **Insulin** | **6(5)** | **Serious** | **Not serious** | **Serious** | **Serious** | **None** | **0.07 (-0.40 to 0.53)** | ⨁◯◯◯ **VERY LOW** |
| **LDL** | **11(10)** | **Serious** | **Serious** | **Serious** | **Serious** | **None** | **-0.50 (-1.01 to 0.00)** | ⨁◯◯◯ **VERY LOW** |
| **HDL** | **12(11)** | **Serious** | **Serious** | **Serious** | **Serious** | **None** | **0.10 (-0.45 to 0.64)** | ⨁◯◯◯ **VERY LOW** |
| **TC** | **11(10)** | **Serious** | **Serious** | **Serious** | **Serious** | **Publication Bias** | **-0.69 (-1.08 to -0.30)** | ⨁◯◯◯ **VERY LOW** |
| **TG** | **11(10)** | **Serious** | **Serious** | **Serious** | **Serious** | **None** | **-0.56 (-0.91 to -0.22)** | ⨁◯◯◯ **VERY LOW** |
| **CRP** | **6(6)** | **Serious** | **Serious** | **Serious** | **Serious** | **None** | **-0.88 (-2.13 to 0.36)** | ⨁◯◯◯ **VERY LOW** |
| **Bone Health Outcomes** | | | | | | | | |
| **leg bone mineral density** | **3(3)** | **Serious** | **Not serious** | **Serious** | **Serious** | **None** | **0.06 (-0.77 to 0.89)** | ⨁◯◯◯ **VERY LOW** |
| **BMD** | **7(7)** | **Serious** | **Not serious** | **Serious** | **Serious** | **None** | **0.22 (-0.07 to 0.52)** | ⨁◯◯◯ **VERY LOW** |
| **leg bone mineral content** | **3(3)** | **Serious** | **Not serious** | **Serious** | **Serious** | **None** | **0.16 (-0.67 to 1.00)** | ⨁◯◯◯ **VERY LOW** |
| **BMC** | **6(6)** | **Serious** | **Not serious** | **Serious** | **Serious** | **None** | **0.22 (-0.11 to 0.55)** | ⨁◯◯◯ **VERY LOW** |
| **plasma osteocalcin** | **4(3)** | **Serious** | **Not serious** | **Serious** | **Not serious** | **None** | **1.07 (0.53 to 1.60)** | ⨁⨁◯◯ **LOW** |
| **P1NP** | **4(3)** | **Serious** | **Serious** | **Serious** | **Not serious** | **None** | **1.28 (0.22 to 2.34)** | ⨁⨁◯◯ **LOW** |
| **CTX-1** | **4(3)** | **Serious** | **Serious** | **Serious** | **Serious** | **None** | **0.54 (-0.49 to 1.58)** | ⨁◯◯◯ **VERY LOW** |
| *** Certainty of evidence according to Grading of Recommendations, Assessment, Development and Evaluations (GRADE):**  **High: We are very confident in the estimated effect**  **Moderate: Our confidence in the estimated effect is moderate**  **Low: We have limited confidence in the estimated effect**  **Very low: We have very little confidence in the estimated effect** | | | | | | | | |

**Note:** GRADE assessment criteria: a. Risk of bias: Downgraded when the contributing studies were predominantly judged as having some concerns or higher risk of bias according to RoB 2 or ROBINS-I. b. Inconsistency: Downgraded when substantial between-study heterogeneity was present, based on the magnitude of I², variability in effect estimates, and/or prediction intervals. c. Indirectness: Downgraded because pooled estimates combined clinically heterogeneous populations with different chronic diseases, treatments, baseline characteristics, and potential responsiveness to exercise. d. Imprecision: Downgraded when confidence intervals were wide, crossed the null or included both clinically important benefit and no effect, and/or when the available information size was limited. e. Publication bias: Downgraded when funnel-plot asymmetry and/or Egger’s regression, where applicable, suggested possible small-study effects; these judgments were interpreted cautiously when the number of contributing studies was small.

BMI = body mass index; WC: waist circumference; SBP: systolic blood pressure; DBP: diastolic blood pressure; MAP: mean arterial pressure; TC: total cholesterol; LDL: low-density lipoprotein cholesterol; HDL: high-density lipoprotein cholesterol; TG: triglycerides; CRP: C-reactive protein; RHR, resting heart rate; YYIE1(m), Yo-Yo Intermittent Endurance Test level 1 performance; BMD: whole body bone mineral density; BMC: whole body bone mineral content; P1NP: procollagen type I N-terminal propeptide; CTX-1: C-terminal telopeptide of type I collagen

| **Table S3 PRISMA 2020 checklist** | | | |
| --- | --- | --- | --- |
| **Section and Topic** | **Item #** | **Checklist item** | **Location where item is reported** |
| **TITLE** | | |  |
| Title | 1 | Identify the report as a systematic review. | Title |
| **ABSTRACT** | | |  |
| Abstract | 2 | See the PRISMA 2020 for Abstracts checklist. | Abstract |
| **INTRODUCTION** | | |  |
| Rationale | 3 | Describe the rationale for the review in the context of existing knowledge. | 1. Introduction |
| Objectives | 4 | Provide an explicit statement of the objective(s) or question(s) the review addresses. | 1. Introduction |
| **METHODS** | | |  |
| Eligibility criteria | 5 | Specify the inclusion and exclusion criteria for the review and how studies were grouped for the syntheses. | 2. Methods — Eligibility criteria |
| Information sources | 6 | Specify all databases, registers, websites, organisations, reference lists and other sources searched or consulted to identify studies. Specify the date when each source was last searched or consulted. | 2. Methods — Search strategy |
| Search strategy | 7 | Present the full search strategies for all databases, registers and websites, including any filters and limits used. | 2. Methods — Search strategy; Table S1 |
| Selection process | 8 | Specify the methods used to decide whether a study met the inclusion criteria of the review, including how many reviewers screened each record and each report retrieved, whether they worked independently, and if applicable, details of automation tools used in the process. | 2. Methods — Selection process |
| Data collection process | 9 | Specify the methods used to collect data from reports, including how many reviewers collected data from each report, whether they worked independently, any processes for obtaining or confirming data from study investigators, and if applicable, details of automation tools used in the process. | 2. Methods — Data extraction |
| Data items | 10a | List and define all outcomes for which data were sought. Specify whether all results that were compatible with each outcome domain in each study were sought (e.g. for all measures, time points, analyses), and if not, the methods used to decide which results to collect. | 2. Methods — Eligibility criteria; Data extraction |
|  | 10b | List and define all other variables for which data were sought (e.g. participant and intervention characteristics, funding sources). Describe any assumptions made about any missing or unclear information. | 2. Methods — Data extraction |
| Study risk of bias assessment | 11 | Specify the methods used to assess risk of bias in the included studies, including details of the tool(s) used, how many reviewers assessed each study and whether they worked independently, and if applicable, details of automation tools used in the process. | 2. Methods — Risk of bias and methodological quality |
| Effect measures | 12 | Specify for each outcome the effect measure(s) (e.g. risk ratio, mean difference) used in the synthesis or presentation of results. | 2. Methods — Statistical analysis |
| Synthesis methods | 13a | Describe the processes used to decide which studies were eligible for each synthesis (e.g. tabulating the study intervention characteristics and comparing against the planned groups for each synthesis (item #5)). | 2. Methods — Eligibility criteria; Statistical analysis |
|  | 13b | Describe any methods required to prepare the data for presentation or synthesis, such as handling of missing summary statistics, or data conversions. | 2. Methods — Data extraction; Statistical analysis |
|  | 13c | Describe any methods used to tabulate or visually display results of individual studies and syntheses. | 2. Methods — Statistical analysis |
|  | 13d | Describe any methods used to synthesize results and provide a rationale for the choice(s). If meta-analysis was performed, describe the model(s), method(s) to identify the presence and extent of statistical heterogeneity, and software package(s) used. | 2. Methods — Statistical analysis |
|  | 13e | Describe any methods used to explore possible causes of heterogeneity among study results (e.g. subgroup analysis, meta-regression). | 2. Methods — Statistical analysis |
|  | 13f | Describe any sensitivity analyses conducted to assess robustness of the synthesized results. | 2. Methods — Statistical analysis |
| Reporting bias assessment | 14 | Describe any methods used to assess risk of bias due to missing results in a synthesis (arising from reporting biases). | 2. Methods — Statistical analysis |
| Certainty assessment | 15 | Describe any methods used to assess certainty (or confidence) in the body of evidence for an outcome. | 2. Methods — Certainty of the evidence |
| **RESULTS** | | |  |
| Study selection | 16a | Describe the results of the search and selection process, from the number of records identified in the search to the number of studies included in the review, ideally using a flow diagram. | 3. Results — Study selection; Fig. 1 |
|  | 16b | Cite studies that might appear to meet the inclusion criteria, but which were excluded, and explain why they were excluded. | 3. Results — Study selection; Fig. 1 |
| Study characteristics | 17 | Cite each included study and present its characteristics. | 3. Results — Characteristics of included studies; Tables 1 and 2 |
| Risk of bias in studies | 18 | Present assessments of risk of bias for each included study. | 3. Results — Risk of bias and certainty of evidence; Figure S1 |
| Results of individual studies | 19 | For all outcomes, present, for each study: (a) summary statistics for each group (where appropriate) and (b) an effect estimate and its precision (e.g. confidence/credible interval), ideally using structured tables or plots. | 3. Results — Effects of recreational football on body composition; Effects on cardiovascular and cardiorespiratory outcomes; Effects on lipid, inflammatory, and glycaemic outcomes; Effects on bone health outcomes; Figs. 2–5 |
| Results of syntheses | 20a | For each synthesis, briefly summarise the characteristics and risk of bias among contributing studies. | 3. Results — Characteristics of included studies; Risk of bias and certainty of evidence; Tables 1 and 2; Figure S1; Table S2 |
|  | 20b | Present results of all statistical syntheses conducted. If meta-analysis was done, present for each the summary estimate and its precision (e.g. confidence/credible interval) and measures of statistical heterogeneity. If comparing groups, describe the direction of the effect. | 3. Results — Outcome-specific effects sections; Effects expressed as mean differences; Figs. 2–5; Tables S6 and S7 |
|  | 20c | Present results of all investigations of possible causes of heterogeneity among study results. | Not reported (subgroup or meta-regression results are not presented). |
|  | 20d | Present results of all sensitivity analyses conducted to assess the robustness of the synthesized results. | 3. Results — Sensitivity analyses; Figures S2–S5; Tables S4–S8 and S10 |
| Reporting biases | 21 | Present assessments of risk of bias due to missing results (arising from reporting biases) for each synthesis assessed. | 3. Results — Outcome-specific effects sections (Egger’s test results); Figs. 2–5 |
| Certainty of evidence | 22 | Present assessments of certainty (or confidence) in the body of evidence for each outcome assessed. | 3. Results — Risk of bias and certainty of evidence; Table S2 |
| **DISCUSSION** | | |  |
| Discussion | 23a | Provide a general interpretation of the results in the context of other evidence. | 4. Discussion — Principal findings; Body composition; Cardiovascular and cardiorespiratory outcomes; Lipid and glycaemic outcomes; Bone outcomes; Mechanistic interpretation; Disease-specific qualitative interpretation |
|  | 23b | Discuss any limitations of the evidence included in the review. | 4. Discussion — Strengths and limitations; Feasibility, adherence, and safety |
|  | 23c | Discuss any limitations of the review processes used. | 4. Discussion — Strengths and limitations |
|  | 23d | Discuss implications of the results for practice, policy, and future research. | 4. Discussion — Clinical interpretation; Feasibility, adherence, and safety; Strengths and limitations; 5. Conclusion |
| **OTHER INFORMATION** | | |  |
| Registration and protocol | 24a | Provide registration information for the review, including register name and registration number, or state that the review was not registered. | Abstract — Trial registration; 2. Methods (opening paragraph) |
|  | 24b | Indicate where the review protocol can be accessed, or state that a protocol was not prepared. | Abstract — Trial registration; 2. Methods (opening paragraph) |
|  | 24c | Describe and explain any amendments to information provided at registration or in the protocol. | N/a |
| Support | 25 | Describe sources of financial or non-financial support for the review, and the role of the funders or sponsors in the review. | Declarations — Funding |
| Competing interests | 26 | Declare any competing interests of review authors. | Declarations — Competing interests |
| Availability of data, code and other materials | 27 | Report which of the following are publicly available and where they can be found: template data collection forms; data extracted from included studies; data used for all analyses; analytic code; any other materials used in the review. | Declarations — Availability of data and materials |

*From:* Page MJ, McKenzie JE, Bossuyt PM, Boutron I, Hoffmann TC, Mulrow CD, et al. The PRISMA 2020 statement: an updated guideline for reporting systematic reviews. BMJ 2021;372:n71. doi: 10.1136/bmj.n71. This work is licensed under CC BY 4.0. To view a copy of this license, visit <https://creativecommons.org/licenses/by/4.0/>

**Table S4 Sensitivity analyses excluding non-randomized controlled trials**

| **Outcome** | **Trials** | **k** | **Primary analysis** | ***p*** | **Excluding non-randomized controlled trials** | ***p*** | **Interpretation** |
| --- | --- | --- | --- | --- | --- | --- | --- |
| Weight | 13 | 13 | −0.29 (−0.58 to 0.00) | 0.051 | −0.32 (−0.65 to 0.01) | 0.055 | Borderline; directionally consistent; concordant under CR2 (p = 0.06) |
| BMI | 8 | 9 | −0.06 (−0.38 to 0.27) | 0.707 | −0.07 (−0.48 to 0.35) | 0.700 | Robust; concordant under CR2 (p = 0.60) |
| VO₂peak | 12 | 16 | 0.70 (0.37 to 1.03) | <0.001 | 0.70 (0.39 to 1.01) | <0.001 | Robust; concordant under CR2 (p < 0.01) |

Note: Sensitivity analyses were conducted after excluding non-randomized controlled trials (CT studies). Two non-randomized trials were eligible (McEwan 2019; Andersen 2014), and refitting was restricted a priori to the three outcomes to which at least two of them contributed; the number of non-randomized trials contributing to every outcome is given in Table S10. All models use the independent trial or participant cohort as the clustering unit (Table S11) and were repeated with cluster-robust (CR2) variance estimation, which gave concordant results for all three outcomes. Trials, the number of contributing independent trials; k, the total number of effect sizes included in the pooled effect size.

| **Table S5. Sensitivity analyses under alternative pre–post correlation assumptions** | | | | | |
| --- | --- | --- | --- | --- | --- |
| **Outcome** | **r = 0.20** | **r = 0.50** | **r = 0.80** | **r = 0.85** | **Interpretation** |
| Weight | g = −0.27 [−0.55, 0.01], p = 0.06 | g = −0.27 [−0.55, 0.01], p = 0.06 | g = −0.28 [−0.57, 0.00], p = 0.05 | g = −0.29 [−0.58, 0.00], p = 0.05 | **Robust borderline finding** |
| BMI | g = −0.03 [−0.34, 0.28], p = 0.83 | g = −0.04 [−0.35, 0.27], p = 0.80 | g = −0.05 [−0.36, 0.27], p = 0.75 | g = −0.06 [−0.38, 0.27], p = 0.71 | **Robust** |
| Body fat% | g = −0.19 [−0.40, 0.02], p = 0.07 | g = −0.23 [−0.44, −0.02], p = 0.04 | g = −0.32 [−0.53, −0.11], p < 0.01 | g = −0.36 [−0.57, −0.14], p < 0.01 | **Non-robust** |
| Fat mass | g = −0.24 [−0.54, 0.05], p = 0.10 | g = −0.25 [−0.55, 0.04], p = 0.08 | g = −0.28 [−0.58, 0.01], p = 0.06 | g = −0.30 [−0.59, 0.00], p = 0.05 | **Non-robust** |
| WC | g = −0.31 [−0.81, 0.19], p = 0.16 | g = −0.34 [−0.87, 0.19], p = 0.15 | g = −0.43 [−1.15, 0.28], p = 0.17 | g = −0.48 [−1.31, 0.35], p = 0.18 | **Robust** |
| Muscle mass | g = 0.16 [−0.05, 0.38], p = 0.13 | g = 0.17 [−0.04, 0.39], p = 0.10 | g = 0.21 [−0.01, 0.42], p = 0.06 | g = 0.22 [0.01, 0.44], p = 0.05 | **Non-robust** |
| SBP | g = −0.41 [−0.84, 0.02], p = 0.06 | g = −0.45 [−0.92, 0.01], p = 0.05 | g = −0.56 [−1.12, −0.01], p = 0.05 | g = −0.60 [−1.20, 0.00], p = 0.05 | **Non-robust** |
| DBP | g = −0.36 [−0.71, −0.02], p = 0.04 | g = −0.40 [−0.77, −0.04], p = 0.03 | g = −0.49 [−0.91, −0.08], p = 0.02 | g = −0.53 [−0.96, −0.09], p = 0.02 | **Robust** |
| MAP | g = −0.63 [−1.03, −0.23], p < 0.01 | g = −0.69 [−1.13, −0.26], p < 0.01 | g = −0.84 [−1.39, −0.28], p = 0.01 | g = −0.89 [−1.48, −0.29], p = 0.01 | **Robust** |
| VO_2_peak | g = 0.37 [0.16, 0.58], p < 0.01 | g = 0.44 [0.20, 0.68], p < 0.01 | g = 0.63 [0.32, 0.94], p < 0.01 | g = 0.70 [0.37, 1.03], p < 0.01 | **Robust** |
| RHR | g = −0.57 [−0.94, −0.19], p < 0.01 | g = −0.65 [−1.07, −0.23], p < 0.01 | g = −0.90 [−1.52, −0.29], p < 0.01 | g = −1.01 [−1.72, −0.31], p = 0.01 | **Robust** |
| YYIE1(m) | g = 1.05 [0.20, 1.90], p = 0.03 | g = 1.13 [0.39, 1.88], p = 0.02 | g = 1.35 [0.76, 1.95], p < 0.01 | g = 1.43 [0.83, 2.03], p < 0.01 | **Robust** |
| HbA1c | g = −0.13 [−0.49, 0.22], p = 0.39 | g = −0.17 [−0.52, 0.19], p = 0.29 | g = −0.26 [−0.61, 0.10], p = 0.13 | g = −0.28 [−0.63, 0.08], p = 0.10 | **Robust** |
| Glucose | g = −0.05 [−0.33, 0.23], p = 0.69 | g = −0.06 [−0.34, 0.22], p = 0.64 | g = −0.09 [−0.39, 0.21], p = 0.51 | g = −0.11 [−0.44, 0.22], p = 0.48 | **Robust** |
| TG | g = −0.33 [−0.59, −0.08], p = 0.01 | g = −0.38 [−0.64, −0.13], p < 0.01 | g = −0.51 [−0.81, −0.21], p < 0.01 | g = −0.56 [−0.91, −0.22], p < 0.01 | **Robust** |
| TC | g = −0.38 [−0.62, −0.13], p < 0.01 | g = −0.45 [−0.69, −0.20], p < 0.01 | g = −0.62 [−0.95, −0.28], p < 0.01 | g = −0.69 [−1.08, −0.30], p < 0.01 | **Robust** |
| HDL | g = 0.04 [−0.20, 0.28], p = 0.75 | g = 0.06 [−0.27, 0.38], p = 0.71 | g = 0.09 [−0.39, 0.58], p = 0.69 | g = 0.10 [−0.45, 0.64], p = 0.70 | **Robust** |
| LDL | g = −0.26 [−0.51, −0.02], p = 0.04 | g = −0.31 [−0.58, −0.04], p = 0.03 | g = −0.45 [−0.87, −0.03], p = 0.04 | g = −0.50 [−1.01, 0.00], p = 0.05 | **Non-robust** |
| CRP | g = −0.53 [−1.31, 0.25], p = 0.14 | g = −0.63 [−1.55, 0.29], p = 0.14 | g = −0.82 [−2.00, 0.35], p = 0.13 | g = −0.88 [−2.13, 0.36], p = 0.13 | **Robust** |
| Insulin | g = 0.03 [−0.41, 0.48], p = 0.85 | g = 0.04 [−0.40, 0.49], p = 0.82 | g = 0.13 [−0.46, 0.72], p = 0.60 | g = 0.07 [−0.40, 0.53], p = 0.73 | **Robust** |
| Bone density of legs | g = 0.03 [−0.81, 0.86], p = 0.90 | g = 0.03 [−0.80, 0.87], p = 0.88 | g = 0.05 [−0.78, 0.89], p = 0.80 | g = 0.06 [−0.77, 0.89], p = 0.78 | **Robust** |
| BMD | g = 0.12 [−0.18, 0.41], p = 0.37 | g = 0.14 [−0.16, 0.43], p = 0.29 | g = 0.20 [−0.10, 0.49], p = 0.15 | g = 0.22 [−0.07, 0.52], p = 0.11 | **Robust** |
| Leg bone mineral content | g = 0.07 [−0.76, 0.90], p = 0.75 | g = 0.09 [−0.74, 0.92], p = 0.69 | g = 0.14 [−0.69, 0.98], p = 0.54 | g = 0.16 [−0.67, 1.00], p = 0.49 | **Robust** |
| BMC | g = 0.14 [−0.18, 0.47], p = 0.31 | g = 0.16 [−0.17, 0.49], p = 0.27 | g = 0.20 [−0.13, 0.53], p = 0.18 | g = 0.22 [−0.11, 0.55], p = 0.15 | **Robust** |
| Plasma osteocalcin | g = 0.53 [0.02, 1.04], p = 0.04 | g = 0.66 [0.14, 1.17], p = 0.03 | g = 0.96 [0.43, 1.49], p = 0.01 | g = 1.07 [0.53, 1.60], p < 0.01 | **Robust** |
| P1NP | g = 0.63 [0.12, 1.14], p = 0.03 | g = 0.78 [0.18, 1.38], p = 0.03 | g = 1.16 [0.22, 2.09], p = 0.03 | g = 1.28 [0.22, 2.34], p = 0.03 | **Robust** |
| CTX-1 | g = 0.22 [−0.28, 0.73], p = 0.25 | g = 0.29 [−0.30, 0.89], p = 0.22 | g = 0.48 [−0.43, 1.38], p = 0.19 | g = 0.54 [−0.49, 1.58], p = 0.19 | **Robust** |
| **Note:** r refers to the assumed pre-post correlation coefficient used to estimate change-score standard deviations. Values are presented as Hedges’ g with 95% confidence intervals. Statistical interpretation was determined according to whether the 95% confidence interval included the null value of 0, rather than the rounded p value. “Robust” indicates that the direction and statistical interpretation were unchanged across all four correlation assumptions. “Robust borderline finding” indicates that the result remained borderline across assumptions, with confidence intervals close to the null value. “Non-robust” indicates that the statistical interpretation, based on whether the 95% confidence interval included 0, changed across correlation assumptions under either the model-based or the cluster-robust (CR2) procedure; the estimates shown are model-based. | | | | | |

**Table S6. Model-based and cluster-robust (CR2) inference for every pooled standardised mean difference**

| **Outcome** | **k** | **Trials** | **SMD** | **Model-based 95% CI** | **p** | **CR2 95% CI** | **CR2 df** | **CR2 p** | **Concordant** |
| --- | --- | --- | --- | --- | --- | --- | --- | --- | --- |
| Weight | 13 | 13 | −0.29 | −0.58 to 0.00 | 0.051 | −0.58 to 0.00 | 11.4 | 0.052 | Yes |
| BMI | 9 | 8 | −0.06 | −0.38 to 0.27 | 0.707 | −0.28 to 0.17 | 6.0 | 0.573 | Yes |
| Body fat percentage | 13 | 13 | −0.36 | −0.57 to −0.14 | 0.004 | −0.57 to −0.14 | 10.6 | 0.004 | Yes |
| Fat mass | 13 | 12 | −0.30 | −0.59 to 0.00 | 0.049 | −0.59 to 0.00 | 10.6 | 0.053 | No |
| Waist circumference | 5 | 5 | −0.48 | −1.31 to 0.35 | 0.181 | −1.31 to 0.34 | 3.8 | 0.176 | Yes |
| Muscle mass | 12 | 11 | 0.22 | 0.01 to 0.44 | 0.045 | 0.07 to 0.37 | 8.9 | 0.009 | Yes |
| SBP | 12 | 11 | −0.60 | −1.20 to 0.00 | 0.050 | −1.21 to 0.01 | 9.7 | 0.052 | No |
| DBP | 12 | 11 | −0.53 | −0.96 to −0.09 | 0.022 | −0.97 to −0.09 | 9.8 | 0.024 | Yes |
| MAP | 7 | 7 | −0.89 | −1.48 to −0.29 | 0.011 | −1.48 to −0.29 | 5.9 | 0.011 | Yes |
| VO₂peak | 16 | 12 | 0.70 | 0.37 to 1.03 | <0.001 | 0.37 to 1.03 | 10.2 | <0.001 | Yes |
| RHR | 10 | 9 | −1.01 | −1.72 to −0.31 | 0.010 | −1.73 to −0.29 | 8.0 | 0.012 | Yes |
| YYIE1 | 4 | 4 | 1.43 | 0.83 to 2.03 | 0.005 | 0.85 to 2.01 | 3.0 | 0.004 | Yes |
| HbA1c | 7 | 6 | −0.28 | −0.63 to 0.08 | 0.103 | −0.63 to 0.07 | 4.5 | 0.093 | Yes |
| Glucose | 10 | 9 | −0.11 | −0.44 to 0.22 | 0.483 | −0.43 to 0.21 | 7.5 | 0.455 | Yes |
| Triglycerides | 11 | 10 | −0.56 | −0.91 to −0.22 | 0.005 | −0.91 to −0.21 | 8.4 | 0.006 | Yes |
| Total cholesterol | 11 | 10 | −0.69 | −1.08 to −0.30 | 0.003 | −1.08 to −0.29 | 8.8 | 0.003 | Yes |
| HDL | 12 | 11 | 0.10 | −0.45 to 0.64 | 0.698 | −0.45 to 0.65 | 9.9 | 0.698 | Yes |
| LDL | 11 | 10 | −0.50 | −1.01 to 0.00 | 0.049 | −1.02 to 0.01 | 8.9 | 0.052 | No |
| CRP | 6 | 6 | −0.88 | −2.13 to 0.36 | 0.128 | −2.13 to 0.36 | 5.0 | 0.128 | Yes |
| Insulin | 6 | 5 | 0.07 | −0.40 to 0.53 | 0.731 | −0.32 to 0.45 | 3.9 | 0.656 | Yes |
| Leg BMD | 3 | 3 | 0.06 | −0.77 to 0.89 | 0.780 | −0.26 to 0.39 | 1.7 | 0.449 | Yes |
| Whole-body BMD | 7 | 7 | 0.22 | −0.07 to 0.52 | 0.113 | 0.03 to 0.42 | 5.6 | 0.030 | No |
| Leg BMC | 3 | 3 | 0.16 | −0.67 to 1.00 | 0.487 | −0.16 to 0.49 | 1.7 | 0.142 | Yes |
| Whole-body BMC | 6 | 6 | 0.22 | −0.11 to 0.55 | 0.148 | −0.03 to 0.47 | 4.6 | 0.075 | Yes |
| Osteocalcin | 4 | 3 | 1.07 | 0.53 to 1.60 | 0.008 | 0.26 to 1.87 | 1.8 | 0.030 | Yes |
| P1NP | 4 | 3 | 1.28 | 0.22 to 2.34 | 0.031 | −0.17 to 2.74 | 2.0 | 0.063 | No |
| CTX-1 | 4 | 3 | 0.54 | −0.49 to 1.58 | 0.191 | −0.85 to 1.94 | 2.0 | 0.234 | Yes |

Note: Three-level random-effects models (rma.mv, random = ~ 1 | trial/effect) fitted to effect sizes derived under a pre–post correlation of r = 0.85, with a working sampling covariance matrix blocked by trial and imputed at ρ = 0.60. The clustering unit is the independent trial or participant cohort, so that linked reports of the same trial share one identifier and separate trials remain distinct irrespective of authorship (Table S11). Model-based inference uses the t distribution with k − 1 degrees of freedom; cluster-robust inference uses the bias-reduced CR2 sandwich estimator with Satterthwaite degrees of freedom (metafor::robust(…, clubSandwich = TRUE)). Point estimates are identical under the two procedures, so only one estimate is shown. "Concordant" indicates agreement on statistical significance at α = 0.05. k, number of effect sizes; Trials, number of contributing independent trials.

**Table S7. Model-based and cluster-robust (CR2) inference for the pooled mean differences**

| **Outcome** | **k** | **Trials** | **MD** | **Model-based 95% CI** | **p** | **CR2 95% CI** | **CR2 df** | **CR2 p** | **Concordant** |
| --- | --- | --- | --- | --- | --- | --- | --- | --- | --- |
| Weight | 13 | 13 | −1.46 | −2.81 to −0.11 | 0.036 | −2.94 to 0.01 | 7.3 | 0.052 | No |
| Body fat percentage | 13 | 13 | −0.87 | −1.26 to −0.48 | <0.001 | −1.52 to −0.21 | 1.7 | 0.031 | Yes |
| Muscle mass | 12 | 11 | 0.86 | 0.22 to 1.50 | 0.013 | 0.37 to 1.35 | 3.4 | 0.010 | Yes |
| BMI | 9 | 8 | −0.33 | −1.22 to 0.57 | 0.424 | −1.34 to 0.68 | 3.9 | 0.415 | Yes |
| Waist circumference | 5 | 5 | −3.84 | −11.82 to 4.14 | 0.253 | −11.70 to 4.03 | 3.9 | 0.246 | Yes |
| MAP | 7 | 7 | −5.10 | −7.87 to −2.33 | 0.004 | −7.91 to −2.30 | 5.7 | 0.005 | Yes |
| RHR | 10 | 9 | −5.73 | −9.66 to −1.80 | 0.009 | −9.74 to −1.73 | 8.0 | 0.011 | Yes |

Note: Mean differences are expressed in the original units of each outcome (kg for weight and muscle mass; % for body fat; kg·m⁻² for BMI; cm for waist circumference; mmHg for mean arterial pressure; beats·min⁻¹ for resting heart rate). Model specification and cluster-robust procedure as in Table S6.

**Table S8. Sensitivity of every pooled estimate to the assumed within-trial sampling correlation ρ**

| **Outcome** | **k** | **ρ = 0.20** | **ρ = 0.40** | **ρ = 0.60 (primary)** | **ρ = 0.80** | **Largest change in SMD** | **Interpretation** |
| --- | --- | --- | --- | --- | --- | --- | --- |
| Weight | 13 | −0.29 (0.051) | −0.29 (0.051) | −0.29 (0.051) | −0.29 (0.051) | 0.00 | Unchanged |
| BMI | 9 | −0.07 (0.606) | −0.06 (0.676) | −0.06 (0.707) | −0.07 (0.686) | 0.02 | Unchanged |
| Body fat percentage | 13 | −0.36 (0.004) | −0.36 (0.004) | −0.36 (0.004) | −0.36 (0.004) | 0.00 | Unchanged |
| Fat mass | 13 | −0.29 (0.048) | −0.29 (0.048) | −0.30 (0.049) | −0.30 (0.050) | 0.00 | Unchanged |
| Waist circumference | 5 | −0.48 (0.181) | −0.48 (0.181) | −0.48 (0.181) | −0.48 (0.181) | 0.00 | Unchanged |
| Muscle mass | 12 | 0.22 (0.043) | 0.22 (0.044) | 0.22 (0.045) | 0.22 (0.051) | 0.00 | Model-based significance changes |
| SBP | 12 | −0.60 (0.050) | −0.60 (0.050) | −0.60 (0.050) | −0.60 (0.050) | 0.00 | Unchanged |
| DBP | 12 | −0.52 (0.024) | −0.52 (0.023) | −0.53 (0.022) | −0.53 (0.021) | 0.01 | Unchanged |
| MAP | 7 | −0.89 (0.011) | −0.89 (0.011) | −0.89 (0.011) | −0.89 (0.011) | 0.00 | Unchanged |
| VO₂peak | 16 | 0.70 (<0.001) | 0.70 (<0.001) | 0.70 (<0.001) | 0.70 (<0.001) | 0.01 | Unchanged |
| RHR | 10 | −1.01 (0.010) | −1.01 (0.010) | −1.01 (0.010) | −1.01 (0.010) | 0.00 | Unchanged |
| YYIE1 | 4 | 1.43 (0.005) | 1.43 (0.005) | 1.43 (0.005) | 1.43 (0.005) | 0.00 | Unchanged |
| HbA1c | 7 | −0.30 (0.078) | −0.29 (0.091) | −0.28 (0.103) | −0.29 (0.127) | 0.02 | Unchanged |
| Glucose | 10 | −0.11 (0.445) | −0.11 (0.465) | −0.11 (0.483) | −0.11 (0.499) | 0.00 | Unchanged |
| Triglycerides | 11 | −0.55 (0.006) | −0.56 (0.005) | −0.56 (0.005) | −0.57 (0.004) | 0.01 | Unchanged |
| Total cholesterol | 11 | −0.69 (0.003) | −0.69 (0.003) | −0.69 (0.003) | −0.69 (0.003) | 0.00 | Unchanged |
| HDL | 12 | 0.10 (0.698) | 0.10 (0.698) | 0.10 (0.698) | 0.10 (0.698) | 0.00 | Unchanged |
| LDL | 11 | −0.50 (0.049) | −0.50 (0.049) | −0.50 (0.049) | −0.50 (0.049) | 0.00 | Unchanged |
| CRP | 6 | −0.88 (0.128) | −0.88 (0.128) | −0.88 (0.128) | −0.88 (0.128) | 0.00 | Unchanged |
| Insulin | 6 | 0.06 (0.737) | 0.06 (0.733) | 0.07 (0.731) | 0.07 (0.738) | 0.01 | Unchanged |
| Leg BMD | 3 | 0.06 (0.780) | 0.06 (0.780) | 0.06 (0.780) | 0.06 (0.780) | 0.00 | Unchanged |
| Whole-body BMD | 7 | 0.22 (0.113) | 0.22 (0.113) | 0.22 (0.113) | 0.22 (0.113) | 0.00 | Unchanged |
| Leg BMC | 3 | 0.16 (0.487) | 0.16 (0.487) | 0.16 (0.487) | 0.16 (0.487) | 0.00 | Unchanged |
| Whole-body BMC | 6 | 0.22 (0.148) | 0.22 (0.148) | 0.22 (0.148) | 0.22 (0.148) | 0.00 | Unchanged |
| Osteocalcin | 4 | 1.05 (0.009) | 1.06 (0.008) | 1.07 (0.008) | 1.08 (0.009) | 0.03 | Unchanged |
| P1NP | 4 | 1.28 (0.031) | 1.28 (0.031) | 1.28 (0.031) | 1.28 (0.031) | 0.00 | Unchanged |
| CTX-1 | 4 | 0.54 (0.196) | 0.54 (0.194) | 0.54 (0.191) | 0.55 (0.190) | 0.01 | Unchanged |

Note: Cells give the pooled standardised mean difference with the model-based p value in parentheses. "Largest change in SMD" is the difference between the largest and the smallest pooled estimate across the four values of ρ. Only muscle mass changes its model-based statistical interpretation across this range (p = 0.043 at ρ = 0.20 to p = 0.051 at ρ = 0.80); under cluster-robust inference it remains significant at every value of ρ, and no other outcome changes its interpretation under either procedure. ρ influences only the 17 outcomes in which at least one trial contributes more than one effect size; elsewhere the working covariance matrix is diagonal (Table S10).

**Table S9. Sampling correlation implied by the effect sizes that share a control group**

| **Outcome** | **Trial** | **Report** | **Control-group n** | **Effect sizes** | **Implied ρ** |
| --- | --- | --- | --- | --- | --- |
| BMI | T03 | Mohammed 2023 | 10 | 2 | 0.482 |
| SBP | T03 | Mohammed 2021 | 10 | 2 | 0.471 |
| DBP | T03 | Mohammed 2021 | 10 | 2 | 0.501 |
| VO₂peak | T03 | Mohammed 2023 | 10 | 2 | 0.477 |
| VO₂peak | T06 | Uth 2020 | 16 | 2 | 0.673 |
| VO₂peak | T14 | Andersen 2014 | 9 | 2 | 0.516 |
| VO₂peak | T16 | Knoepfli-Lenzin 2010 | 17 | 2 | 0.483 |
| RHR | T03 | Mohammed 2023 | 10 | 2 | 0.573 |
| HbA1c | T03 | Mohammed 2021 | 10 | 2 | 0.503 |
| Glucose | T03 | Mohammed 2021 | 10 | 2 | 0.488 |
| Triglycerides | T03 | Mohammed 2021 | 10 | 2 | 0.501 |
| Total cholesterol | T03 | Mohammed 2021 | 10 | 2 | 0.477 |
| HDL | T03 | Mohammed 2021 | 10 | 2 | 0.518 |
| LDL | T03 | Mohammed 2021 | 10 | 2 | 0.497 |
| Insulin | T03 | Mohammed 2021 | 10 | 2 | 0.488 |
| Osteocalcin | T11 | Uth 2016 | 28 | 2 | 0.520 |
| P1NP | T11 | Uth 2016 | 28 | 2 | 0.516 |
| CTX-1 | T11 | Uth 2016 | 28 | 2 | 0.508 |

Note: For two standardised mean differences computed against a shared control group the sampling covariance is approximately 1/n_C + d₁d₂/(2N), where n_C is the control-group sample size, d₁ and d₂ are the two effect sizes and N is the total sample size of the comparison (Gleser & Olkin, 2009). The implied correlation is that covariance divided by the geometric mean of the two sampling variances. Across the five trials that contribute more than one effect size to a single outcome the implied correlation ranges from 0.47 to 0.67 (median 0.50), bracketing the assumed value of ρ = 0.60.

**Table S10. Effect sizes, contributing trials and contribution of non-randomized controlled trials for each outcome**

| **Outcome** | **Effect sizes (k)** | **Contributing trials** | **Non-randomized trials** | **Non-randomized effect sizes** | **Refitted after exclusion** |
| --- | --- | --- | --- | --- | --- |
| Weight | 13 | 13 | 2 | 2 | Yes |
| BMI | 9 | 8 | 2 | 2 | Yes |
| Body fat percentage | 13 | 13 | 1 | 1 | No |
| Fat mass | 13 | 12 | 1 | 1 | No |
| Waist circumference | 5 | 5 | 0 | 0 | No |
| Muscle mass | 12 | 11 | 1 | 1 | No |
| SBP | 12 | 11 | 1 | 1 | No |
| DBP | 12 | 11 | 1 | 1 | No |
| MAP | 7 | 7 | 0 | 0 | No |
| VO₂peak | 16 | 12 | 2 | 3 | Yes |
| RHR | 10 | 9 | 0 | 0 | No |
| YYIE1 | 4 | 4 | 0 | 0 | No |
| HbA1c | 7 | 6 | 1 | 1 | No |
| Glucose | 10 | 9 | 1 | 1 | No |
| Triglycerides | 11 | 10 | 0 | 0 | No |
| Total cholesterol | 11 | 10 | 0 | 0 | No |
| HDL | 12 | 11 | 0 | 0 | No |
| LDL | 11 | 10 | 0 | 0 | No |
| CRP | 6 | 6 | 1 | 1 | No |
| Insulin | 6 | 5 | 1 | 1 | No |
| Leg BMD | 3 | 3 | 1 | 1 | No |
| Whole-body BMD | 7 | 7 | 0 | 0 | No |
| Leg BMC | 3 | 3 | 1 | 1 | No |
| Whole-body BMC | 6 | 6 | 0 | 0 | No |
| Osteocalcin | 4 | 3 | 0 | 0 | No |
| P1NP | 4 | 3 | 0 | 0 | No |
| CTX-1 | 4 | 3 | 0 | 0 | No |

Note: Two of the 17 independent trials were non-randomized controlled trials (McEwan 2019; Andersen 2014). Models were refitted after excluding them only for the three outcomes to which at least two non-randomized trials contributed (Table S4). Comparison of the "Effect sizes" and "Contributing trials" columns identifies where within-trial dependence exists: k equals the number of trials in 10 outcomes, exceeds it by one in 16 outcomes, and exceeds it by four for VO₂peak.

**Table S11. Assignment of each included report to its underlying trial or participant cohort**

| **Report** | **Trial identifier** | **Linked report(s) from the same trial** | **Design** |
| --- | --- | --- | --- |
| Teixeira et al, 2023 | T01 | — | RCT |
| Poffé et al, 2023 | T02 | — | RCT |
| Mohammed et al, 2023 | T03 | Mohammed et al., 2021 | RCT |
| Soares et al, 2022 | T04 | — | RCT |
| Vasconcellos et al, 2021 | T05 | — | RCT |
| Mohammed et al, 2021 | T03 | Mohammed et al., 2023 | RCT |
| Uth et al, 2020 | T06 | — | RCT |
| McEwan et al, 2019 | T07 | — | Non-randomized |
| Skoradal et al, 2018a | T08 | Skoradal et al., 2018b | RCT |
| Skoradal et al, 2018b | T08 | Skoradal et al., 2018a | RCT |
| Krustrup et al, 2017 | T09 | — | RCT |
| De Sousa et al, 2017 | T10 | — | RCT |
| Uth et al, 2016a | T11 | Uth et al., 2016b; Uth et al., 2014 | RCT |
| Uth et al, 2016b | T11 | Uth et al., 2016a; Uth et al., 2014 | RCT |
| Sousa et al, 2015 | T12 | — | RCT |
| Mohr et al, 2015 | T13 | Mohr et al., 2014 | RCT |
| Uth et al, 2014 | T11 | Uth et al., 2016a; Uth et al., 2016b | RCT |
| Mohr et al, 2014 | T13 | Mohr et al., 2015 | RCT |
| Andersen et al, 2014 | T14 | — | Non-randomized |
| Krustrup et al, 2013 | T15 | — | RCT |
| Knoepfli-Lenzin et al, 2010 | T16 | — | RCT |
| Andersen et al, 2010 | T17 | — | RCT |

Note: The 22 included reports correspond to 17 independent trials or participant cohorts. Reports sharing a trial identifier describe the same participants and are treated as one cluster in every analysis; reports with different identifiers are treated as independent even when they share a first author (for example Uth et al., 2020 versus Uth et al., 2014/2016, and Krustrup et al., 2013 versus Krustrup et al., 2017). This identifier defines the third level of every multilevel model, the blocking of the working covariance matrix, the clustering unit of the CR2 cluster-robust variance estimator, and the unit of exclusion in the leave-one-out analyses.

**Figure S1. Risk-of-bias assessment of the 17 independent trials.** (A) RoB 2 assessment of 15 independent randomized controlled trials. Multiple publications originating from the same underlying trial were consolidated into a single trial-level assessment. (B) ROBINS-I assessment of the two non-randomized controlled trials.

**Figure S2. Sensitivity analysis of body composition outcomes**

**Figure S3. Sensitivity analysis of cardiovascular and cardiorespiratory outcomes**

**Figure S4. Sensitivity analysis of lipid, inflammatory, and glycaemic outcomes**

**Figure S5. Sensitivity analysis of bone health outcomes**
